# High-entropy induced a glass-to-glass transition in a metallic glass

**DOI:** 10.1038/s41467-022-29789-1

**Published:** 2022-04-21

**Authors:** Hengwei Luan, Xin Zhang, Hongyu Ding, Fei Zhang, J. H. Luan, Z. B. Jiao, Yi-Chieh Yang, Hengtong Bu, Ranbin Wang, Jialun Gu, Chunlin Shao, Qing Yu, Yang Shao, Qiaoshi Zeng, Na Chen, C. T. Liu, Ke-Fu Yao

**Affiliations:** 1 School of Materials Science and Engineering, Tsinghua University, 100084 Beijing, China; 2 Center for High Pressure Science and Technology Advanced Research, 201203 Shanghai, China; 3 Marine Equipment and Technology Institute, Jiangsu University of Science and Technology, 212003 Zhenjiang, China; 4 State Key Laboratory for Advanced Metals and Materials, University of Science and Technology Beijing, 100083 Beijing, China; 5 Department of Materials Science and Engineering, City University of Hong Kong, 999077 Hong Kong, China; 6 Department of Mechanical Engineering, The Hong Kong Polytechnic University, 999077 Hong Kong, China; 7 School of Mathematical Sciences, Peking University, 100871 Beijing, China; 8 Department of Mechanical Engineering, City University of Hong Kong, 999077 Hong Kong, China; 9 Hong Kong Institute of Advanced Study (HKIAS) and College of Engineering, City University of Hong Kong, 999077 Hong Kong, China

**Keywords:** Glasses, Metals and alloys

## Abstract

Glass-to-glass transitions are useful for us to understand the glass nature, but it remains difficult to tune the metallic glass into significantly different glass states. Here, we have demonstrated that the high-entropy can enhance the degree of disorder in an equiatomic high-entropy metallic glass NbNiZrTiCo and elevate it to a high-energy glass state. An unusual glass-to-glass phase transition is discovered during heating with an enormous heat release even larger than that of the following crystallization at higher temperatures. Dramatic atomic rearrangement with a short- and medium-range ordering is revealed by in-situ synchrotron X-ray diffraction analyses. This glass-to-glass transition leads to a significant improvement in the modulus, hardness, and thermal stability, all of which could promote their applications. Based on the proposed high-entropy effect, two high-entropy metallic glasses are developed and they show similar glass-to-glass transitions. These findings uncover a high-entropy effect in metallic glasses and create a pathway for tuning the glass states and properties.

## Abstract (web summary)

Glass-to-glass transitions can help understanding the glass nature, but it remains difficult to tune metallic glasses into significantly different glass states. Here the authors demonstrate the high-entropy effects in glass-to-glass transitions of high-entropy metallic glasses.

## Introduction

Among the material world, metallic glasses (MGs) constitute an attractive and unusual class of advanced materials for fundamental studies and structural applications. Their amorphous structure without a long-range periodicity renders MGs with very unique and excellent properties^1–4^ and tunable glass states^5–7^ with different atomic structures and energies. The exothermic signal detected by calorimetry provides the most straightforward method to characterize the energy states of glasses^8^, which is closely related to their structures and properties^9,10^. However, typical structural relaxation in MGs only involves limited local atom position adjustment with small heat release before the crystallization^11,12^.

Compared with the local structural relaxation mediated transitions of different glass states, glass-to-glass phase transitions of MGs could tune the glass states and energies more significantly, but they are rarely observed. An extremely high pressure has been reported as a possible method to introduce the phase transition in some MGs, but the high-pressure-state MGs cannot be maintained at an ambient pressure for further characterization^13,14^, and they are also limited to particular compositions with elements prone to pressure-induced electronic transitions. Recent literature reported that the reentrant glass transition and the hidden amorphous phase existed in ternary Pd-Ni-P alloys^15,16^. The presence of anomalous exothermic peaks (AEPs) in their supercooled liquid regions was attributed to the change of atomic structure, while the detailed thermodynamic mechanism and structural change remain to be discovered. To gain an in-depth understanding and harness the glass-to-glass transition, it is highly desirable to discover a pathway to access distinct MGs through glass-to-glass transitions with a significant structural change and property improvement.

Besides enthalpy, entropy, a fundamental thermodynamic parameter and the measurement of disorder, is also proposed to play an important role in the glass formation by affecting the atomic packing^17^. However, the entropy is seldom introduced as an effective tool to control the glass states in practice^18^. The main reasons are the relatively small contribution of entropy to the energy and the difficulty in harnessing it in conventional MGs. This might be overcome by the emergence of high-entropy alloys (HEAs) with equal or near-equal mole fractions of five or more constituent elements in alloy compositions^19^. With a continuously increasing research interest in HEAs^20,21^, the concept of the high-entropy has quickly spread into diverse material systems^22,23^. The high-entropy metallic glasses (HEMGs), with amorphous structures and HEA-like compositions, not only expand the composition space for searching MGs, but also have already shown unique properties, such as high crystallization resistance and excellent mechanical properties^24–30^. However, the effect of high-entropy on the glass structures and glass-to-glass transition remains to be resolved.

In this work, we have demonstrated that the high-entropy can effectively enhance the degree of the disorder in an equiatomic NbNiZrTiCo HEMG and enables the formation of a very high-energy glass state. This high-energy glass can transform into a stable low-energy glass via a glass-to-glass transition with an enormous exotherm even larger than that of its crystallization.

## Results

### The glass-to-glass transition and the glass structures of the HEMG

**Fig. 1:**
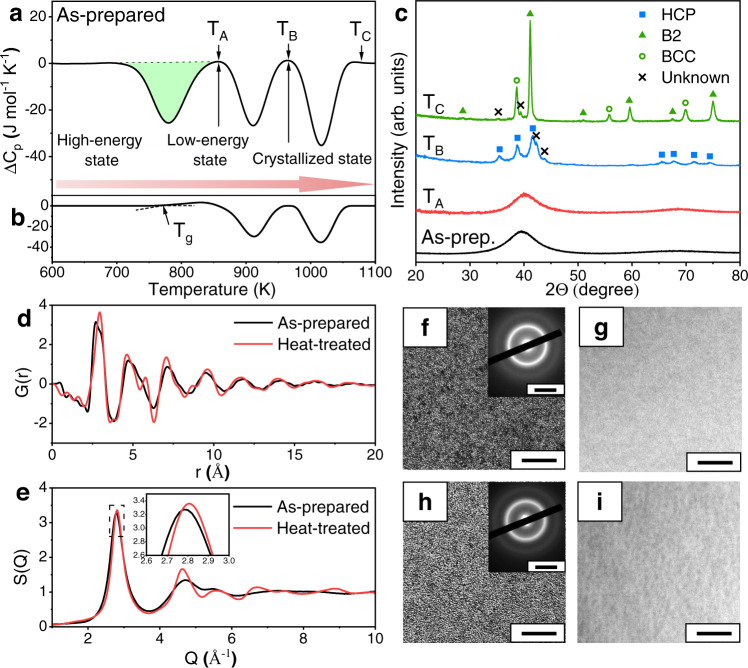
Specific heat (Δ*C*_P_), X-ray diffraction, synchrotron X-ray diffraction, HRTEM, SAED, and HAADF analyses of the as-prepared and heat-treated NbNiZrTiCo HEMGs. **a** DSC curve of the as-prepared sample. The structures of the alloy are denoted and the anomalous exothermic peak (filled in green) shows heat release comparable to that of crystallization. During heating, the HEMG undergoes phase transitions from a high-energy glass state to a low-energy glass state and further to a crystallized state (red arrow). **b** DSC curve of the heat-treated sample. The first exothermic peak in the as-prepared sample disappears. To highlight the phase transitions, the curve of the crystallized HEMG (samples heated to 1173 K) was subtracted in **a** and **b**. **c** XRD results of the as-prepared and heat-treated samples. The *T*_A_, *T*_B_, and *T*_C_ denote the heat treatment temperatures. As-prep.: As-prepared sample. **d** Synchrotron X-ray diffraction results of the as-prepared and heat-treated samples in real space measured at room temperature. **e** Synchrotron X-ray diffraction results of the as-prepared and heat-treated samples in *Q*-space measured at room temperature. Inset: enlarged first diffraction peak in the dotted box. **f** HRTEM (scale bar: 5 nm) and SAED (inset, scale bar: 5 nm^−1^) result of the as-prepared sample. **g** HAADF (scale bar: 20 nm) result of the as-prepared sample. **h**–**i** Same as **f**–**g** but for the heat-treated sample. The results show the heat-treated sample remains amorphous with different glass structure and no crystallization or detectable compositional inhomogeneity. Additional HRTEM results are in Supplementary Note 3.

Figure 1a displays the obtained differential scanning calorimetry (DSC) scan curve of the as-prepared NbNiZrTiCo alloy, which has an amorphous structure as evidenced by the X-ray diffraction (XRD) (the As-prep. curve in Fig. 1c). Three exothermic peaks are observed at 781, 911, and 1016 K in the DSC curve with released heats of 1.65, 1.27, and 1.59 kJ mol^−1^, respectively. No obvious glass transition temperature (*T*_g_) is observed in the conventional DSC curve of the as-prepared sample. The *T*_g_ of the as-prepared sample can be observed before the first exothermic peak with heating rates higher than 1000 K s^−1^ by Flash DSC (Supplementary Note 1 and Supplementary Fig. 7). DSC analyses with multiple heating rates are also performed (10, 20, 30, 40, and 50 K min^−1^, including the heating rate for the in-situ diffraction experiments shown later), and the activation energies corresponding to the three exothermic peaks are obtained to be 281, 384, and 249 kJ mol^−1^, respectively (Supplementary Note 2 and Supplementary Fig. 8). To investigate the structural evolution of the HEMG underlying each exothermic peak, the as-prepared HEMG is heated to the temperatures just above each exothermic peak (*T*_A_: 855 K, *T*_B_: 963 K, and *T*_C_: 1076 K, respectively, as shown in Fig. 1a), and then cooled down to room temperature for further characterization. Surprisingly, the sample heated to *T*_A_ (denoted as heat-treated sample hereinafter) remains amorphous (see the corresponding XRD pattern denoted as *T*_A_ in Fig. 1c). A hexagonal close-packed (HCP) structure is formed after heated to *T*_B_ (*T*_B_ curve in Fig. 1c), while a dual-phase structure of compositional disordered body-centered cubic (BCC) and ordered BCC (B2) is obtained after heated to *T*_C_ (*T*_C_ curve in Fig. 1c). It should be noted that some weak diffraction peaks (marked with x) on the spectra of the samples heated to *T*_B_ and *T*_C_ cannot be well identified due to their low intensities and the limited number of peaks. They are considered not significant enough because the current work is mainly focused on the glass-to-glass transition below *T*_A_.

Based on the above experimental results, it can be concluded that the exothermic reaction corresponding to the first exothermic peak on the DSC curve does not lead to the crystallization, although its heat release is even larger than that of the following crystallization. Since both the as-prepared sample and heated-treated sample are glasses (*T*_g_ in Supplementary Note 1 and Fig. 1b), it is indicated that the as-prepared sample experiences a glass-to-glass transition from a high-energy state to a low-energy state with a large energy reduction. The second exothermic peak corresponds to crystallization, and the third exothermic peak corresponds to a crystalline-to-crystalline transition.

To further investigate the structural differences associated with the transition, the synchrotron X-ray diffraction is applied on the as-prepared and heat-treated samples at room temperature. The as-prepared sample shows a typical reduced pair-distribution function *G*(*r*) (As-prepared curve in Fig. 1d), and a total structure function *S*(*Q*) (as-prepared curve in Fig. 1e) of MGs with no sharp Bragg diffraction pattern. The amorphous structure is also confirmed by the high-resolution transmission electron microscopy (HRTEM) and selected area electron diffraction (SAED) results (Fig. 1f, more images in Supplementary Note 3 and Supplementary Figs. 9, 10, 11, 12), which show a uniform amorphous structure and a typical diffraction pattern of MGs without nanocrystals. Figure 1g shows the high-angle annular dark-field (HAADF) image acquired in the scanning transmission electron microscopy (STEM) mode for the as-prepared sample. The HAADF image is sensitive to the atomic number of the constituent elements of the sample, and the acquired image shows no clear contrast, indicating that the element distribution is homogeneous at the nanometer scale.

As for the heat-treated sample, Fig. 1b shows its DSC curve where only the latter two exothermic peaks can be observed. The first exothermic peak noticed in the as-prepared sample disappears while a clear glass transition signal can be observed with a glass transition temperature (*T*_g_) at ~764 K, which indicates that the glass-to-glass transition corresponding to the first exothermic peak is completed and irreversible. Synchrotron X-ray diffraction results show that the heat-treated sample possesses an amorphous structure, but its *G*(*r*) (heat-treated curve in Fig. 1d) at room temperature is quite different from that of the as-prepared sample. Specifically, the intensity maximum moves from the left side (as-prepared curve in Fig. 1d) to the right side (heat-treated curve in Fig. 1d) of the first peak in *G*(*r*). Moreover, a clearer peak splitting is noticed in the second *G*(*r*) peak of the HEMG after heat treatment. These changes observed in *G*(*r*) suggest a significant short- and medium-range structural change, which is quite different from that of the typical structural relaxation^31–37^. As for the *S*(*Q*) (Fig. 1e), slight peak position shifting and sharpening of the first peak can be observed with a clearer splitting in the second peak, which reflects a structural ordering of the short- and medium-range structures as well^38–41^. Synchrotron small-angle X-ray scattering (SAXS) experiments are further performed and confirm the amorphous structure (Supplementary Note 4 and Supplementary Fig. 13). The HRTEM and SAED results (Fig. 1h) further confirm the heat-treated sample remains amorphous with no crystalline phase, which is confirmed by additional HRTEM analyses (Supplementary Note 3), while the HAADF image (Fig. 1i) shows slight contrast. During isothermal annealing experiments, the amorphous structure of the heat-treated sample remains stable after annealed at *T*_g_ – 5 K (759 K) for 2 h and even at *T*_g_ + 30 K (794 K) for 2 or 10 h (Supplementary Note 5 and Supplementary Fig. 14), respectively, which demonstrates the excellent thermal stability of the low-energy MG. Based on these results, an irreversible glass-to-glass transition from a high-energy HEMG (the as-prepared sample) to a low-energy HEMG (the heat-treated sample) is discovered, which is accompanied by an enormous heat release and a significant short- and medium-range structural change.

### Atom probe tomography (APT) analyses

**Fig. 2:**
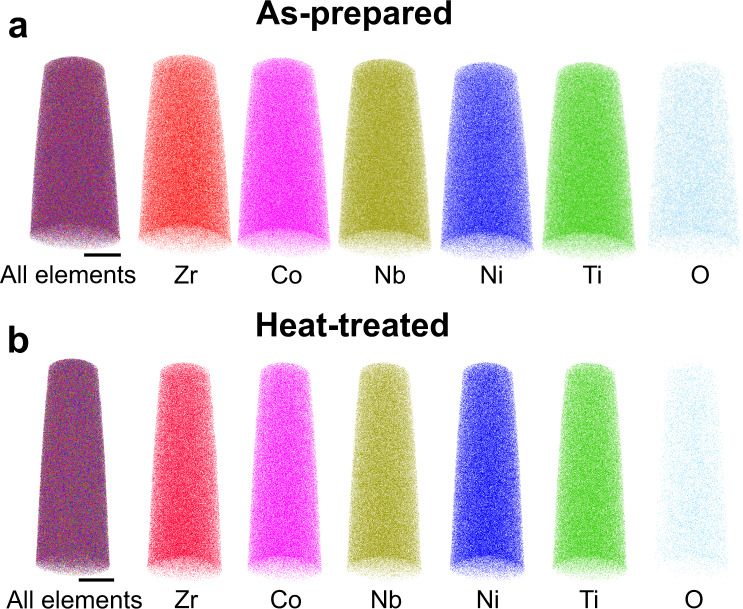
APT analyses of the as-prepared and heat-treated samples. **a** The APT results of the as-prepared sample. **b** The APT results of the heat-treated sample. The results show no compositional heterogeneity. Scale bar: 20 nm. Additional APT results and quantitative analyses in Supplementary Note 6.

Figure 2a, b display the APT results of the as-prepared and heat-treated samples, respectively. Both samples show uniform element distribution within a sub-nanometer scale. Besides, the APT results show a low oxygen content (<0.4 at.%) in the samples, which could be introduced during the sample preparation process or from the chamber of the APT tests. Together with the HAADF results, it is suggested that no compositional inhomogeneity is detected in either nanometer or sub-nanometer scales before or after the glass-to-glass transition, excluding the possibility of spinodal decomposition. These results are further confirmed by additional APT measurements and quantitative analyses (Supplementary Note 6, Supplementary Figs. 15, 16, Supplementary Table 2). However, the detailed structural change cannot be detected due to the limited spatial resolution of the APT.

### In-situ high-temperature synchrotron X-ray diffraction

**Fig. 3:**
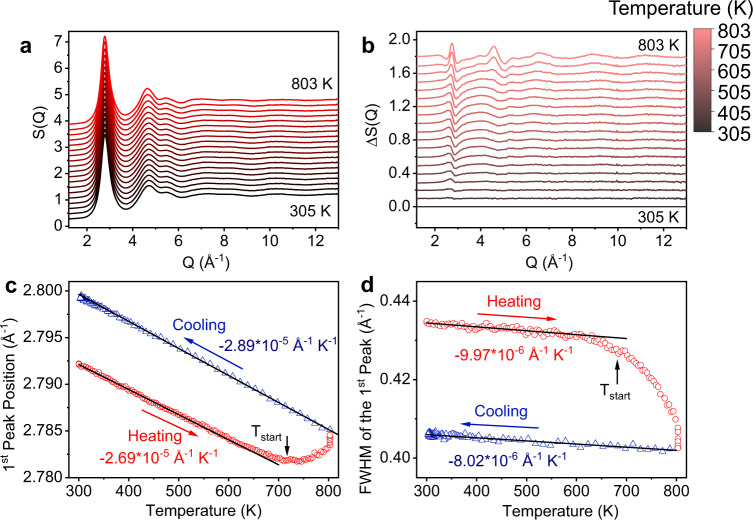
In-situ X-ray diffraction results of the NbNiZrTiCo HEMG in *Q*-space. **a** Evolution of structure function *S*(*Q*) of the as-prepared sample during heating. **b** The respective differences in structure functions obtained by subtracting the reference pattern at *T* = 305 K. **c** The evolution of the first peak position of *S*(*Q*) as a function of temperature. **d** The evolution of the full width at half maximum (FWHM) of the first peak as a function of temperature. The start temperature of the first exothermic peak in the 30 K min^−1^ DSC curve is denoted as *T*_start_ in **c** and **d**. The first peak shows irreversible shifting towards high angle and narrowing.

In-situ synchrotron X-ray diffraction is applied to reveal the structure evolution of the HEMG through the glass-to-glass transition during heating. Figure 3a shows the evolution of the *S*(*Q*) of the sample during continuous heating with a heating rate of 30 K min^−1^ from room temperature (305 K) to 803 K, which is close to the upper-temperature limit of the heating furnace and above the onset temperature of the first exothermic peak (denoted as *T*_start_ at 717 K, obtained from the 30 K min^−1^ DSC curve in Supplementary Fig. 8). To highlight the structural change, the difference curves Δ*S*(*Q*) are plotted in Fig. 3b, where the *S*(*Q*) at 305 K is subtracted as a reference. As the temperature increases, the first peak in *S*(*Q*) shows a continuously linear shift to the low-*Q* direction. When the temperature is close to *T*_start_, the shift of the peak position deviates from the linear variation steeply (Supplementary Fig. 4), and even turns backward to high-*Q* direction. During the later cooling process, the position of the first peak of *S*(*Q*) shifts to the high-*Q* part linearly but with a slightly different slope (Fig. 3c, *S*(*Q*) curve during cooling in Supplementary Fig. 1). Integration of the | Δ*S*(*Q*) | (Supplementary Fig. 3) provides the overall peak changes as a function of temperature, which shows a linear increase at low temperatures with an accelerated change from ~*T*_start_. Moreover, a significant decrease of the full width at half maximum (FWHM) is observed (Fig. 3d). The narrowing of the first peak indicates that the sample enters a more ordered glass state^42^. Overall, a significant non-linear and irreversible peak position and shape change of the first peak in *S*(*Q*) is observed during the glass-to-glass transition, which is consistent with the changes observed on the heat-treated sample measured at room temperature (Fig. 1e).

**Fig. 4:**
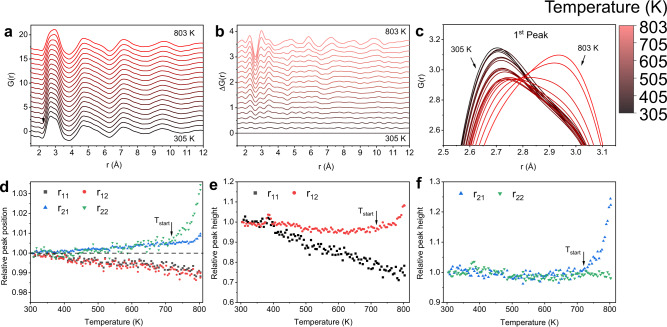
In-situ X-ray diffraction results in real space. **a** Evolution of the reduced pair-distribution functions *G*(*r*) of the as-prepared sample during heating. **b** The respective differences in the reduced pair-distribution functions obtained by subtracting the reference pattern at *T* = 305 K. **c** The enlarged image of the evolution of the first peak as a function of temperature. **d** Evolution of the peak positions of the first and second peaks as a function of temperature. **e** Evolution of the peak intensities of the sub-peaks of the first peak as a function of temperature. **f** Evolution of the peak intensities of the sub-peaks of the second peak as a function of temperature. The start temperature of the first exothermic peak in the 30 K min^−1^ DSC curve is denoted as *T*_start_. Both peaks are fitted with two Gaussian functions.

To obtain more detailed information of the structure evolution in real space, the reduced pair-distribution function curve, *G*(*r*), is derived from *S*(*Q*) with Fourier transform (Fig. 4a). The differential curves Δ*G*(*r*) where the *G*(*r*) at 305 K is subtracted as the reference (Fig. 4b, *G*(*r*) curves during cooling are shown in Supplementary Fig. 2). To highlight the local structural change, the enlarged image of the first peak is shown in Fig. 4c. The most obvious change is the asymmetric shape change in the first peak of *G*(*r*), which shows an intensity maximum on the left side at low temperatures and gradually shifts to the right side at high temperatures. More quantitative information of the local structural change can be obtained by fitting the peak positions of *G*(*r*). For this five-component alloy, there are fifteen atomic pairs with overlapped peaks in the *G*(*r*) curve, which hinder the separation of these atomic pairs. To obtain a realistic analysis, a practical method is to employ minimized numbers of Gaussian functions to obtain a good fitting of the *G*(*r*) peak regardless of the multiple atomic pairs^43–45^. For this sample, two Gaussian sub-peaks, *r*_11_ and *r*_12,_ can fit the first peak of *G*(*r*) very well with a coefficient of determination (*R*^2^) better than 0.999 (Supplementary Note 7 and Supplementary Fig. 17). As the temperature increases, the relative changes of the peak positions and peak heights of *r*_11_ and *r*_12_ are shown in Fig. 4d, e, respectively, by normalized to the corresponding values at 305 K. In Fig. 4d, it is found that *r*_11_ and *r*_12_ consistently shift to the low-*r* part as the temperature increases over the entire temperature range. This peak position contraction contradicts the normal thermal expansion and indicates a significant short- and medium-range structural change. In Fig. 4e, the peak height of *r*_11_ decreases significantly, while the peak height of *r*_12_ slightly decreases and then increases after *T*_start_. The inconsistent variation of the peak heights of the two sub-peaks causes the dramatic shape change of the first peak in *G*(*r*) (Fig. 4c). A similar method is also applied to the second peak of *G*(*r*) to quantify its peak splitting (Fig. 4d,  f). Before *T*_start_, the peak positions and peak heights of the *r*_21_ and *r*_22_ peaks change consistently during heating. After *T*_start_, the bifurcations of both the peak positions and peak heights of *r*_21_ and *r*_22_ can be clearly noticed, which suggest that the splitting of the second peak of *G*(*r*) becomes prominent. These results indicate that the glass-to-glass transition is associated with significant short- and medium-range structural changes, and the changes in the short-range structures is much stronger than typical structural relaxation^31–37^ and glass-to-glass or liquid-to-liquid transitions^15,16,46^. Based on these results, a schematic structural change of the phase transition is prepared (Supplementary Note 7 and Supplementary Fig. 18).

Accompanying the structural change, significant changes in the mechanical properties are also observed by the dynamic mechanical analysis (DMA) (Supplementary Note 8 and Supplementary Fig. 19) and nanoindentation (Supplementary Note 9 and Supplementary Fig. 20 and Supplementary Table 3) measurements. Both measurements show a significantly increased modulus and the nanoindentation result shows a drastic increase of ~40% in the hardness from 6.78 to 9.46 GPa after the glass-to-glass transition, suggesting the formation of stronger atomic bonds in the low-energy MG after structural changes involved in the glass-to-glass transition.

## Discussions

**Fig. 5:**
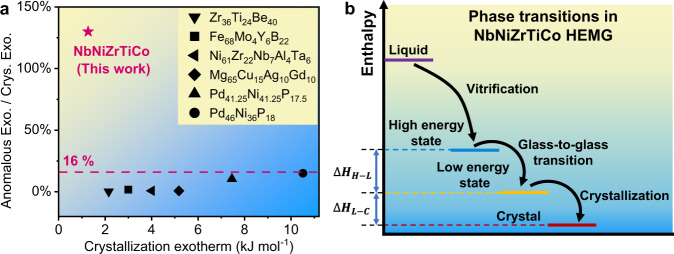
The comparison with other MGs with AEPs and the schematic diagram of phase transitions. **a** The ratios of the anomalous exotherm to the crystallization exotherm of NbNiZrTiCo and other typical MGs with AEPs^15,71–75^. Anomalous Exo./Crys. Exo.: Anomalous exotherm/crystallization exotherm. **b** Upon fast cooling, the liquid could form a high-energy glass and transform into a low-energy glass by glass-to-glass transition and releasing energy $(\Delta {H}_{{{H}}-{{L}}})$. The heat-treated glass could crystallize by further releasing energy $(\Delta {H}_{{{L}}-{{C}}})$.

Compared to other MGs with AEPs, the heat release of the AEP in NbNiZrTiCo HEMG is very large as shown in Fig. 5a. It can be noticed that the typical anomalous exotherm of other reported MGs is usually less than 16% of the crystallization exotherm, while the exotherm of NbNiZrTiCo HEMG is ~130% of the following crystallization exotherm, resulting in a unique glass-to-glass transition. The value of this exotherm is also much larger than the energy increase by thermal cycling^47^. To show the dynamic and thermodynamic pictures of this glass-to-glass transition, Fig. 5b illustrates the temperature dependence of the enthalpy of the NbNiZrTiCo HEMG at a constant pressure. It has been observed that the crystallization can be avoided upon cooling below the freezing point *T*_m_, if the liquid is cooled sufficiently fast (10^4^–10^6^ K s^−1^ in melt-spinning). The “confusion principle” and the Inoue criteria point out that the more elements involved, the lower the chance that the alloy can select viable crystal structures and the easier for the formation of glasses^18,48^. As a result, the formation of most MGs requires two or more elements and the monoatomic MG is rare to make^49^. The reason behind this is that the increased number of elements increases the entropy by enlarging the configuration space, and may cause a sluggish diffusion effect^50–53^ by hindering the configurational sampling. Hence, the glass may not reach a low-energy state dynamically because the atomic reconstruction is limited during the fast vitrification process. This mechanism is expected to be applicable to the selection of glass states as the more elements involved, the lower is the chance for the alloy to access low-energy glass states within a limited time window during quenching. In other words, for a given high-entropy alloy, the high cooling rate and the high-entropy effect can dynamically stabilize the high-energy melt^54,55^. As the measurement of disorder, entropy thus bridges the atomic structure in terms of the atomic packing and its energy state of a given alloy system. How the high-entropy induces the unusual glass-to-glass transition is the key to a deeper understanding of the high-entropy effect in glass formation.

**Fig. 6:**
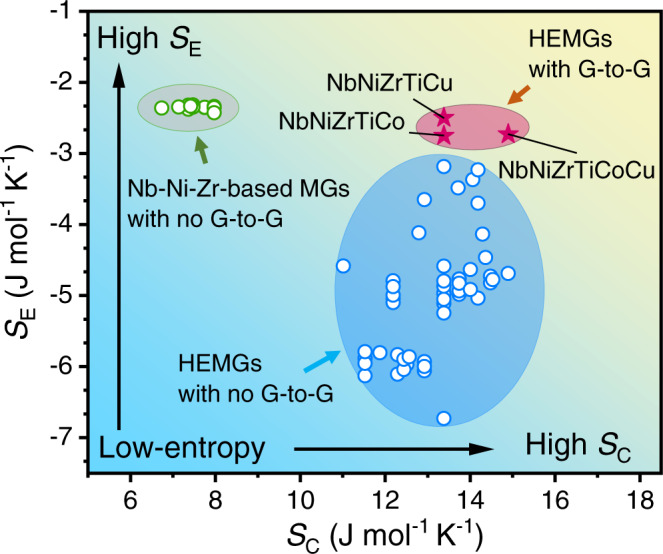
*S*_E_–*S*_C_ plot. The *S*_C_ and *S*_E_ of the HEMGs with glass-to-glass transition, HEMGs without glass-to-glass transition, and the Nb–Ni–Zr-based MGs. The HEMGs with glass-to-glass transition shows high *S*_C_ and *S*_E_. G-to-G: glass-to-glass transition.

**Fig. 7:**
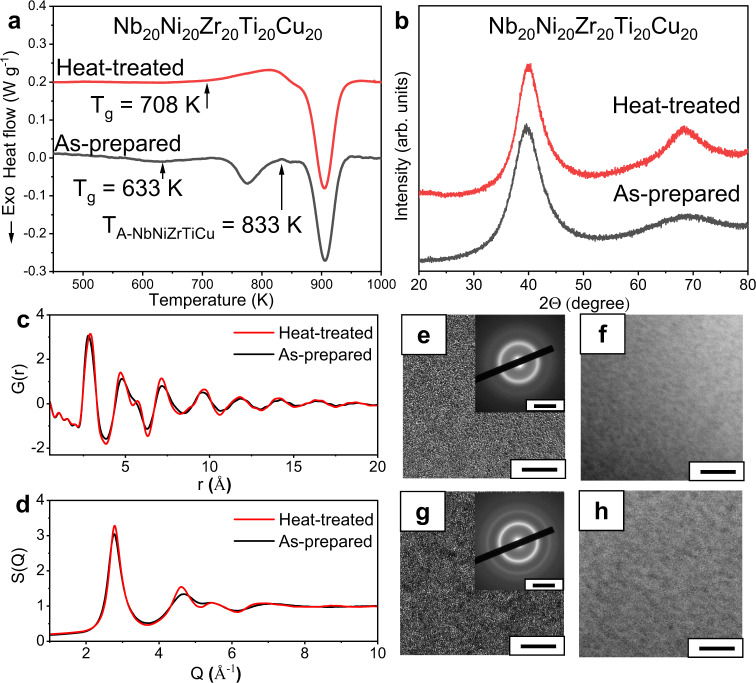
DSC curve, X-ray diffraction, synchrotron X-ray diffraction, HRTEM, SAED, and HAADF analyses of the as-prepared and heat-treated NbNiZrTiCu HEMGs. **a** DSC curve of the as-prepared and heat-treated sample. The as-prepared sample shows a *T*_g_ of 633 K. After heated to *T*_A-NbNiZrTiCu_ = 833 K, the previous first exothermic peak disappears and the heat-treated sample shows a *T*_g_ of 708 K. To highlight the phase transitions, the curve of the crystallized HEMG (samples heated to 1173 K) was subtracted. **b** XRD results of the as-prepared and heat-treated samples. **c** Synchrotron X-ray diffraction results of the as-prepared and heat-treated samples in real space measured at room temperature. **d** Synchrotron X-ray diffraction results of the as-prepared and heat-treated samples in *Q*-space measured at room temperature. **e** HRTEM (scale bar: 5 nm) and SAED (inset, scale bar: 5 nm^−1^) result of the as-prepared sample. **f** HAADF (scale bar: 20 nm) result of the as-prepared sample. **g**, **h** Same as **e**, **f** but for the heat-treated sample. The results show the heat-treated sample remains amorphous with different glass structures and no crystallization or detectable compositional inhomogeneity. Additional HRTEM results are in Supplementary Note 3.

**Fig. 8:**
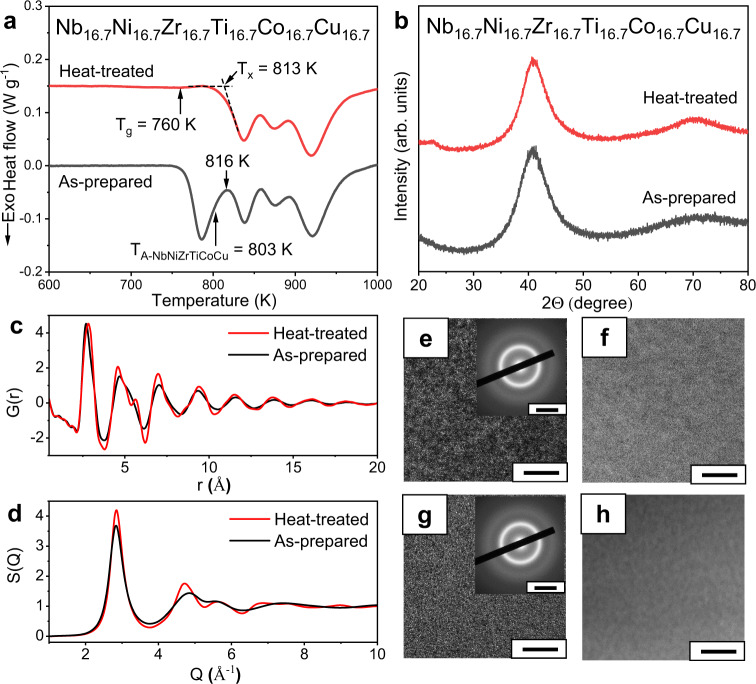
DSC curve, X-ray diffraction, synchrotron X-ray diffraction, HRTEM, SAED, and HAADF analyses of the as-prepared and heat-treated NbNiZrTiCoCu HEMGs. **a** DSC curve of the as-prepared and heat-treated sample. After heated to *T*_A-NbNiZrTiCoCu_ = 803 K, the previous first exothermic peak disappears and the heat-treated sample shows a *T*_g_ of 708 K and *T*_x_ of 813 K. To highlight the phase transitions, the curve of the crystallized HEMG (samples heated to 1173 K) was subtracted. It should be noted that the first two exothermal peaks (corresponding to the glass-to-glass transition and crystallization in the NbNiZrTiCoCu HEMG, respectively) are not completely separated but slightly overlap in the DSC curve. Such peak overlapping results in a partial crystallization when the sample was heated above the first exothermic peak (816 K) (Supplementary Fig. 5), while the sample still remains amorphous when heated to 803 K, which is below the crystallization temperature (813 K, denoted as *T*_x_). **b** XRD results of the as-prepared and heat-treated samples. **c** Synchrotron X-ray diffraction results of the as-prepared and heat-treated samples in real space measured at room temperature. **d** Synchrotron X-ray diffraction results of the as-prepared and heat-treated samples in *Q*-space measured at room temperature. **e** HRTEM (scale bar: 5 nm) and SAED (inset, scale bar: 5 nm^−1^) result of the as-prepared sample. **f** HAADF (scale bar: 20 nm) result of the as-prepared sample. **g**, **h** Same as **e**, **f** but for the heat-treated sample. The results show that the heat-treated sample remains amorphous with different glass structures and no crystallization or detectable compositional inhomogeneity. Additional HRTEM results are in Supplementary Note 3.

The major configurational entropy term of the MGs and HEAs is considered as the configurational entropy of mixing for an ideal solution (denoted as *S*_C_)^21^. First, a comparison of the *S*_C_ for the conventional low *S*_C_ Nb–Ni–Ti-based MGs (denoted by green circles) and the NbNiZrTiCo HEMG (denoted by a red star) has been performed in Fig. 6. Compared to the NbNiZrTiCo HEMG, none of the conventional low *S*_C_ Nb–Ni–Zr-based BMGs shows any exceptional exothermic peak or glass-to-glass transition^56^. Therefore, it is reasonable to assume that the high *S*_C_ in the NbNiZrTiCo HEMG plays an important role in inducing the glass-to-glass transition, provided that these MGs contain the same constituent elements. However, to our best knowledge, such glass-to-glass transition has never been reported in other HEMGs so far. This indicates that the high *S*_C_ alone may not be sufficient for inducing the glass-to-glass transition. In fact, the total configurational entropy of mixing *S*_T_ of an alloy can be written as *S*_T_ = *S*_C_ + *S*_*E*_, where *S*_E_ denotes the excess configurational entropy of mixing, which excludes the contribution from an ideal solution and can be related to the atomic size misfit in the atomic packing^57–59^. Although all HEMGs have more or less high *S*_C_, the *S*_E_ of some HEMGs can be rather low, and thus these HEMGs do not have high *S*_T_. For example, the Sr_20_Ca_20_Yb_20_Mg_20_Zn_20_ HEMG^60^ shows a *S*_E_ of −6.73 J mol^−1^ K^−1^, while the NbNiZrTiCo HEMG in this work shows a *S*_E_ of −2.75 J mol^−1^ K^−1^. Although the two HEMGs have the same *S*_C_ of 13.38 J mol^−1^ K^−1^, they have significantly different *S*_T_ of 6.65 and 10.63 J mol^−1^ K^−1^, respectively, where the *S*_T_ of the NbNiZrTiCo HEMG is ~60 % higher. A comparison of the *S*_E_ of the previously reported HEMGs (denoted by blue circles) and the present NbNiZrTiCo HEMG is also performed in Fig. 6. It is found that the previously reported HEMGs form a low *S*_E_ region, whereas the NbNiZrTiCo HEMG deviates from this region by showing a higher *S*_E_ than those without glass-to-glass transition. This indicates that the excess configurational entropy also plays an important role in inducing the glass-to-glass transition and further confirms the high-entropy effect. Combining the above results, it can be concluded that the high-entropy indeed induces the glass-to-glass transition. The importance of *S*_E_ can also be seen from the perspective of atomic packing and glass formation. *S*_E_ is related to the atomic size misfit and the atomic packing fraction^57–59^, which are the key factors in the phase formation of solid solutions, amorphous phases or intermetallic compounds and their phase stabilities, especially the glass formation of the MGs^18^. The lower the value of *S*_E_, the larger the atomic size difference and usually the higher the atomic packing density^61^. As a result, the MGs with lower *S*_E_ usually show higher glass-forming ability and are expected to form conventional low-energy glasses with high atomic packing fractions^62,63^. The conventional glasses are usually stable, and thus similar phase transition is rare. To experimentally confirm the high-entropy effect, we substitute the Co with Cu in the NbNiZrTiCo HEMG because the Co and Cu have similar atomic radiuses (125 and 128 pm^64^, respectively), and thus the NbNiZrTiCo and NbNiZrTiCu HEMGs have similar high *S*_E_ (−2.75 and −2.50 J mol^−1^ K^−1^, respectively), and the NbNiZrTiCu HEMG still keeps the high *S*_C_ (13.38 J mol^−1^ K^−1^) as shown in Fig. 6 (denoted by a red star). As expected, the NbNiZrTiCu HEMG indeed shows a glass-to-glass transition (Fig. 7), similar to that of the NbNiZrTiCo HEMG. To further confirm the high-entropy effect, a six-element NbNiZrTiCoCu HEMG is developed (*S*_C_: 14.90 J mol^−1^ K^−1^, *S*_E_: −2.73 J mol^−1^ K^−1^) by adding Cu to the NbNiZrTiCo HEMG. As expected, this alloy also shows the glass-to-glass transition as expected (Fig. 8). The *S*_C_, *S*_E_, and *S*_T_ of these MGs are listed in Supplementary Table 1. These experimental results further support the proposed high-entropy effect in inducing the glass-to-glass transition. The resulted low-energy glasses also show increased modulus and hardness by nanoindentation (Supplementary Note 10, Supplementary Table 4), as well as excellent thermal stabilities (Supplementary Note 11, Supplementary Fig. 21). These additional results further suggest that a high-entropy effect is discovered, which enables a wide tunability of the glass states, and indicate that such glass-to-glass transitions would not be limited in the present HEMGs. Compared to the previous works on MGs with metalloid elements (e.g., P^15,16^) or rare earth elements (e.g., La, Ce^46^), the proposed high-entropy effect moves the phenomenon from the special cases to general cases with a series of HEMGs discovered, instead of only one special MG. In such consideration, the HEMGs may serve as model materials in exploring the glass-to-glass transitions and glass nature in the fields of materials science and condensed-matter physics.

In conclusion, we discovered an unusual glass-to-glass transition in a NbNiZrTiCo HEMG with a large exotherm. It was found that the high-entropy effect could dynamically elevate the MG to a high-energy glass state and thermodynamically enable the glass-to-glass transition from the high-energy glass state to a low-energy glass state during heating. Through this simple and non-destructive heat-treating process, this glass-to-glass transition can alter the modulus and hardness more remarkably than rejuvenation by thermal cycling^47^ or mechanical deformation^65^. Besides, the HEMGs might also be applied as promising energetic materials, where the large anomalous exotherm may contribute to their combustion heats and lower the ignition temperatures^66^. In addition, the obtained low-energy state HEMGs exhibit excellent thermal stability. The present results provide an opportunity for further tuning the glass structure and property and addressing the fundamental issues of MGs.

## Methods

### Sample preparation

The NbNiZrTiCo, NbNiZrTiCu, and NbNiZrTiCoCu ingots were prepared by arc melting the mixtures of pure Nb, Ni, Zr, Ti, Co, and Cu (purity ≥ 99.9 wt.%) under a high-purity argon atmosphere. The ingots were flipped and remelted five times to ensure their homogeneity. Ribbons with a thickness of ~45 μm were produced by melt-spinning method with a single-copper roller at a wheel surface velocity of 32 m s^−1^ under a high-purity argon atmosphere.

### Differential scanning calorimetry (DSC)

DSC curves were measured using Al_2_O_3_ crucibles with a synchronous thermal analyzer (STA-449 F3, NETZSCH, Germany) under a high-purity argon atmosphere. The weight of each tested sample was ~20 mg and the heating rate was 20 K min^−1^. For the heat-treated samples, the heat treatment was conducted by a tube furnace and the heating rate and cooling rate were both 20 K min^−1^. The sample was sealed in a glass tube under a high-purity argon atmosphere to avoid oxidation. The temperature of the sample in the furnace was calibrated by a K-type thermocouple thermometer (DT-3891G, CEM, China) heated together with the sample. The thermocouple was inside another tube with the opening blocked by an aluminum foil ball, and the tube itself was identical to the tube containing the sample. The sample and the thermocouple were set in the furnace as close as possible. The temperature of the sample in the furnace was calibrated by the thermocouple, which could compensate the temperature lags. To confirm that the temperature lag of sample heat-treated in the furnace has been excluded, we compared the DSC curves of the sample heat-treated in the furnace and the sample heat-treated in the DSC (Supplementary Fig. 24). The two DSC curves show consistent results and confirm that the temperature lag has been excluded. To highlight the phase transitions, the DSC curve of the crystallized HEMG (samples heated to 1173 K) was subtracted.

### X-ray diffraction (XRD)

The XRD experiments were performed using an X-ray diffractometer (D/max-RB, Rigaku Inc., Japan) with Cu *Kα* radiation (wavelength 1.5406 Å) at room temperature. The XRD experiments were performed using a *θ–2θ* mode with a scanning rate of 3 degrees min^−1^.

### Transmission electron microscopy (TEM) related analyses

TEM samples were ground by 2000 mesh sandpaper to ~30 μm in thickness and then thinned by an ion mill (Gatan 695, U.S.). The samples were first ion milled at 5 keV, 5°/6° for ~4 h, followed by 3 keV, 2°/3° for ~20 min. The high-resolution transmission electron microscopy (HRTEM) measurements and high-angle annular dark-field (HAADF) measurements under scanning transmission electron microscopy (STEM) mode were performed by a high-resolution transmission electron microscopy (JEM-2100F, JEOL, Japan) at 200 kV. The probe size in STEM mode was ~1.5 nm.

### Atom probe tomography (APT) analyses

APT analyses were applied to the as-prepared and heat-treated samples to investigate the element distribution in three dimensions up to a sub-nanometer spatial resolution. Needle-shaped specimens required for APT were fabricated by lift-outs and annular milled in a FEI Scios focused ion beam/scanning electron microscope (FIB/SEM). The APT characterizations were performed in a local electrode atom probe (CAMECA LEAP 5000 XR). The specimens were analyzed at 65 K in voltage mode with a pulse repetition rate of 200 kHz, a pulse fraction of 20%, and an evaporation detection rate of 0.3% atom per pulse. The spatial resolution of the APT could reach ~0.2 nm. Imago Visualization and Analysis Software (IVAS) version 3.8 was used for creating the 3D reconstructions and data analyses.

### Synchrotron X-ray diffraction and pair-distribution functions analyses

In-situ heating high-energy synchrotron X-ray diffraction experiments were conducted at the beamline 11-IDC of the Advanced Photon Source (APS), Argonne National Laboratory (ANL). The wavelength of the X-ray beam was 0.1173 Å (105.711 keV). The beam size was 0.2 × 0.2 mm. Diffraction was performed in a transmission mode by using a two-dimensional (2D) Perkin Elmer amorphous silicon detector with 2048 × 2048 pixels and pixel size of 200 × 200 μm^2^. Ten layers of ribbon samples were folded up to increase the sample thickness and enhance the scattering signal. The samples were tightly sandwiched with two Cu sheets with a tiny hole at the center, and then fixed on the silver heater by a ring clam in the Linkam furnace. For continuous heating and cooling experiments, the Linkam THMS600 heating oven was used to heat the samples from 305 K (room temperature) to 803 K (530 °C) with a heating rate of 30 K min^−1^ and a cooling rate of 50 K min^−1^ to room temperature after annealed at 803 K for 1 min. All patterns were collected with a total exposure time of 10 s, which consists of 10 sub-frames with an exposure time of 1 s for each frame to avoid detector saturation. Representative 2D diffraction images are shown in Supplementary Fig. 6.

The synchrotron X-ray diffraction results of the as-prepared and heat-treated samples were obtained at room temperature. The synchrotron X-ray diffraction of the heat-treated NbNiZrTiCo sample was performed at the beamline 13-IDD of APS, ANL. The wavelength of the X-ray beam was 0.3344 Å (37.0 keV). The beam size was 3.5 × 2 μm. Diffraction was performed in a transmission mode by using a 2D Pilatus 300 K detector with 1043 × 981 pixels and a pixel size of 172 × 172 μm^2^. The synchrotron X-ray diffraction of other samples were performed at the beamline 11-IDD of APS, ANL. The wavelength of the X-ray beam was 0.1173 Å (105.711 keV). The beam size was 0.2 × 0.2 mm. Diffraction was performed in a transmission mode by using a 2D Perkin Elmer amorphous silicon detector with 2048*2048 pixels and pixel size of 200 × 200 μm^2^.

The one-dimensional diffraction patterns were obtained by integrating the 2D diffraction images using Fit2D software^67^. The total scattering factor *S*(*Q*) ($Q=4\pi \,\sin \theta /\lambda$, where *λ* is the X-ray wavelength and *2θ* is the angle between diffraction beam and incident beam)^68^, and the reduced pair-distribution functions *G*(*r*) were obtained using PDFgetX3 software package^69^. To show the reliability of the PDFgetX3 software in this work, we have compared the diffraction results of the as-prepared NbNiZrTiCo sample processed by the PDFgetX3, PDFgetX2 and GSASII softwares, and find that the use of PDFgetX3 software does not have a significant influence on the shapes and the peak positions of the result (Supplementary Note 12 and Supplementary Figs. 22 and 23). The peak positions and the FWHMs of the first *S*(*Q*) peak were obtained by fitting the Voigt function. The total structure function *S*(*Q*) was obtained from the integrated intensities ${I}^{{{{{{\rm{coh}}}}}}}(Q)$ after the correction of Laue diffuse scattering and normalization to the atomic X-ray form factor ${{\langle\, f^{2}\rangle }}$ by

1 $$S(Q)=1\,+\,\frac{{I}^{{{{{{\rm{coh}}}}}}}(Q)-\langle \,{f}^{2}\rangle }{{\langle\, f\rangle }^{2}}$$

The *G*(*r*) function was obtained from the sine-Fourier transform of the total scattering factor *S*(*Q*) by

2 $$G(r)=4\pi r[\rho (r)-{\rho }_{0}]=\frac{2}{\pi }{\int }_{0}^{\infty }Q[S(Q)-1]\,\sin (Qr)dQ$$

where *ρ*(*r*) is the pair density functions and *ρ*_*0*_ is the average number density.

### Calculations of the entropy, enthalpy, and energy

The quantitative difference in enthalpy between the two glasses can be obtained from the DSC curve as $\Delta {H}_{H-L}=-1.65\;{{{{{\mathrm{kJ mo}}}}}}{{{{{{\mathrm{l}}}}}}}^{-1}$. The lower limit of the difference in entropy can be obtained from the DSC curve by

3 $${\Delta}S_{\rm H-L}={\int} dS\ge\int\,-\,\frac{đQ}{T}$$

where d*S* is the infinitesimal entropy change, đ*Q* is the infinitesimal heat release during the phase transition, *T* is the absolute temperature, and the integration range is from the start temperature to the end temperature of the phase transition. This integration results in $\Delta {S}_{{{H}}-{{L}}}=-1.96\;{{{{{\mathrm{J mo}}}}}}{{{{{{\mathrm{l}}}}}}}^{-1}{{{{{{\mathrm{ K}}}}}}}^{-1}$, which is the lower limit of the entropy change because the observed phase transition is an irreversible process. The upper limit difference in Gibbs free energy can be obtained by $\Delta {G}_{H-L}=\Delta {H}_{H-L}-T\Delta {S}_{H-L}$, which is a function of temperature and gives −1.06, −0.24, −0.12, and 0.03 kJ mol^−1^ at 300 K, the start temperature of the first exothermic peak (*T*_start_, 717 K), the peak temperature of the first exothermic peak (781 K) and the end temperature of the first exothermic peak (*T*_A_, 855 K), respectively. It should be emphasized that the negative $\Delta {H}_{H-L}$ and negative $\Delta {S}_{{{H}}-{{L}}}$ are not contradictory to the proposed high-entropy effect. The high-entropy effect elevates the HEMG to a high-energy glass state, which allows the following transition.

The *S*_C_ can be given by the regular solution model widely applied to high-entropy alloys^21^ by

4 $${S}_{{{{{{\mathrm{C}}}}}}}=-R\mathop{\sum }\limits_{i=1}^{m}{x}_{i}\,{{{{\mathrm{ln}}}}}({x}_{i})$$

where *x*_*i*_ is the mole fraction of element *i*, *m* is the number of elements and *R* is the ideal gas constant. For the studied equiatomic NbNiZrTiCo HEMG with five elements, this model gives ${S}_{{{{{{\mathrm{C}}}}}}}=13.38\;{{{{{\mathrm{J mo}}}}}}{{{{{{\mathrm{l}}}}}}}^{-1}{{{{{{\mathrm{ K}}}}}}}^{-1}$.

The *S*_E_ can be given by the Carnahan and Starling theory^58,59^ by

5 $$\frac{{S}_{{{{{{\mathrm{E}}}}}}}}{{k}_{B}}=\frac{(F-{F}^{id})}{{k}_{B}T}-\,{{{{\mathrm{ln}}}}}(Z)-(3-2\xi ){(1-\xi )}^{-2}+3+\,{{{{\mathrm{ln}}}}}\,[(1+\xi +{\xi }^{2}-{\xi }^{3}){(1-\xi )}^{-3}]$$

in which:

6 $$\frac{(F-{F}^{id})}{{k}_{B}T}=	\, -\frac{3}{2}(1-{y}_{1}+{y}_{2}+{y}_{3})+(3{y}_{2}+2{y}_{3}){(1-\xi )}^{-1}\\ 	+\frac{3}{2}\left(1-{y}_{1}-{y}_{2}-\frac{1}{3}{y}_{3}\right){(1-\xi )}^{-2}+(\,{y}_{3}-1){{{{\mathrm{ln}}}}}(1-\xi )$$

7 $$Z=[(1+\xi +{\xi }^{2})-3\xi (\,{y}_{1}+{y}_{2}\xi )-{\xi }^{3}{y}_{3}]{(1-\xi )}^{-3}$$

8 $${y}_{1}=\mathop{\sum }\limits_{j > i=1}^{m}{\varDelta }_{ij}({d}_{i}+{d}_{j}){({d}_{i}{d}_{j})}^{-1/2}$$

9 $${y}_{2}=\mathop{\sum }\limits_{j > i=1}^{m}{\varDelta }_{ij}\mathop{\sum }\limits_{k=1}^{m}\left(\frac{{\xi }_{k}}{\xi }\right)\frac{{({d}_{i}{d}_{j})}^{1/2}}{{d}_{k}}$$

10 $${y}_{3}={\left[\mathop{\sum }\limits_{i=1}^{m}{\left(\frac{{\xi }_{i}}{\xi }\right)}^{2/3}{c}_{i}^{1/3}\right]}^{3}$$

11 $${\varDelta }_{ij}=\frac{{({\xi }_{i}{\xi }_{j})}^{1/2}}{\xi }\frac{{({d}_{i}-{d}_{j})}^{2}}{{d}_{i}{d}_{j}}{({c}_{i}{c}_{j})}^{1/2}$$

12 $${\xi }_{i}=\xi \frac{{d}_{i}^{3}{c}_{i}}{\mathop{\sum }\limits_{j=1}^{m}{d}_{j}^{3}{c}_{j}}$$

where *d*_*i*_ is the atomic diameter of the *i*^th^ element, *c*_*i*_ is the mole fraction of the *i*^th^ element, *ξ* is the atomic packing fraction and *k*_*B*_ is the *Boltzmann constant. The* atomic diameter of metallic elements were taken from the metallic diameters^64^, and the atomic diameter of non-metallic elements were taken from the empirical diameters^70^. The *S*_E_ was computed by averaging the values obtained, respectively, at the atomic packing fraction of 0.68 (BCC-like packing) and 0.74 (FCC-like packing)^58^.

## Supplementary information

Supplementary Information

## Data Availability

The data that support the findings of this study are available within the paper and the Supplementary Information, and all data are available from the authors on reasonable request.
