# Supplementary material for: High-entropy induced a glass-to-glass transition in a metallic glass
Source: Nat Commun. 2022 Apr 21;13:2183. doi: 10.1038/s41467-022-29789-1 (PMC9023469; doi:10.1038/s41467-022-29789-1)
Supplement: Supplementary file 1 — Supplementary Information [file 41467_2022_29789_MOESM1_ESM.docx]

**Supplementary Information**

**High-entropy induced a glass-to-glass transition in a metallic glass**

Hengwei Luan^1†^, Xin Zhang^2†^, Hongyu Ding^1,3†^, Fei Zhang^4,2^, J. H. Luan^5^, Z. B. Jiao^6^, Yi-Chieh Yang^1^, Hengtong Bu^1^, Ranbin Wang^1^, Jialun Gu^1^, Chunlin Shao^7^, Qing Yu^8^, Yang Shao^1*^, Qiaoshi Zeng^2*^, Na Chen^1*^, C.T. Liu^9^, Ke-Fu Yao^1*^

^1^School of Materials Science and Engineering, Tsinghua University, Beijing 100084, China.

^2^Center for High Pressure Science and Technology Advanced Research, Shanghai 201203, China.

^3^Marine Equipment and Technology Institute, Jiangsu University of Science and Technology, Zhenjiang 212003, China.

^4^State Key Laboratory for Advanced Metals and Materials, University of Science and Technology Beijing, Beijing 100083, China.

^5^Department of Materials Science and Engineering, City University of Hong Kong, Hong Kong 999077, China.

^6^Department of Mechanical Engineering, The Hong Kong Polytechnic University, Hong Kong 999077, China.

^7^School of Mathematical Sciences, Peking University, Beijing 100871, China.

^8^Department of Mechanical Engineering, City University of Hong Kong, Hong Kong 999077, China.

^9^Hong Kong Institute of Advanced Study (HKIAS) and College of Engineering, City University of Hong Kong, Hong Kong 999077, China.

†: These authors contributed equally to this work

*: Corresponding authors:

Yang Shao: shaoyang@mail.tsinghua.edu.cn;

Qiaoshi Zeng: zengqs@hpstar.ac.cn;

Na Chen: chennadm@mail.tsinghua.edu.cn;

Ke-Fu Yao: kfyao@mail.tsinghua.edu.cn

**Supplementary Figures**

**
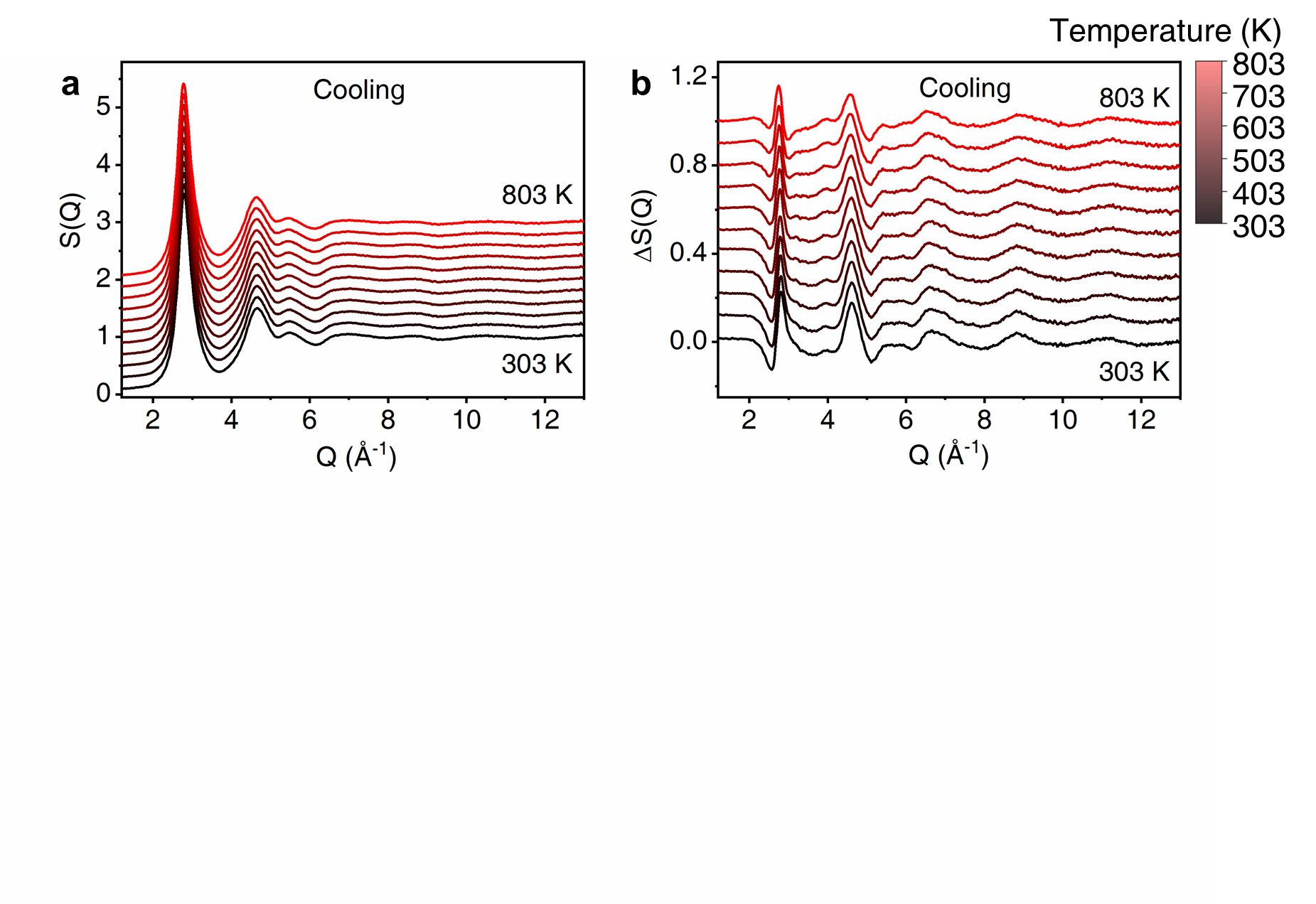
**

**Supplementary Figure 1.** ***In-situ* X-ray diffraction results in *Q*-space during cooling.** **a** Evolution of synchrotron X-ray diffraction pattern of the as-prepared sample during cooling. **b** The respective differences in diffraction patterns obtained by subtracting the reference pattern at *T* = 305 K (before heating).

**
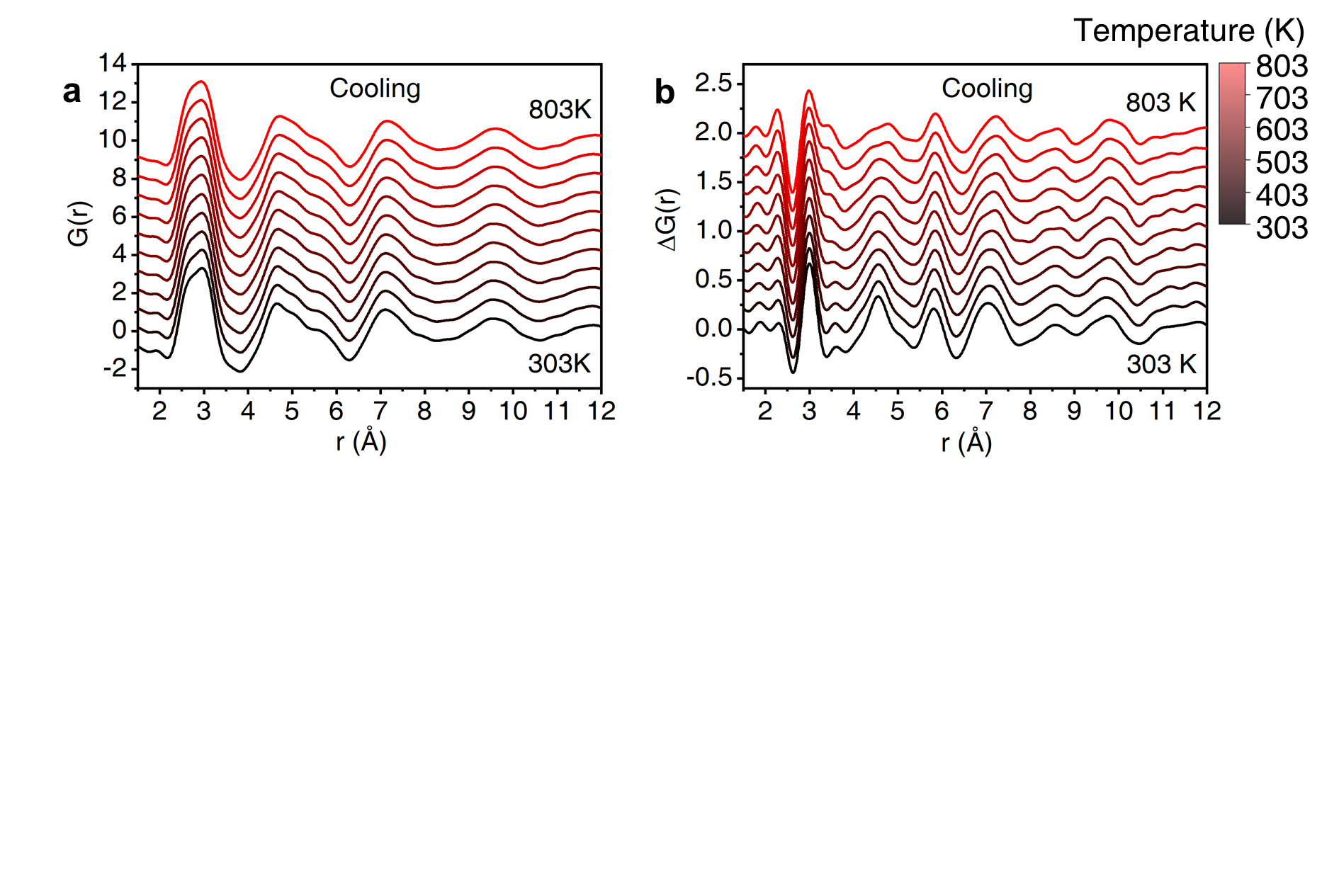
**

**Supplementary Figure 2.** ***In-situ* X-ray diffraction results in real space. a** Evolution of the reduced pair-distribution function of the as-prepared sample during cooling. **b** The respective differences in the reduced pair-distribution functions obtained by subtracting the reference pattern at *T* = 305 K (before heating).

**
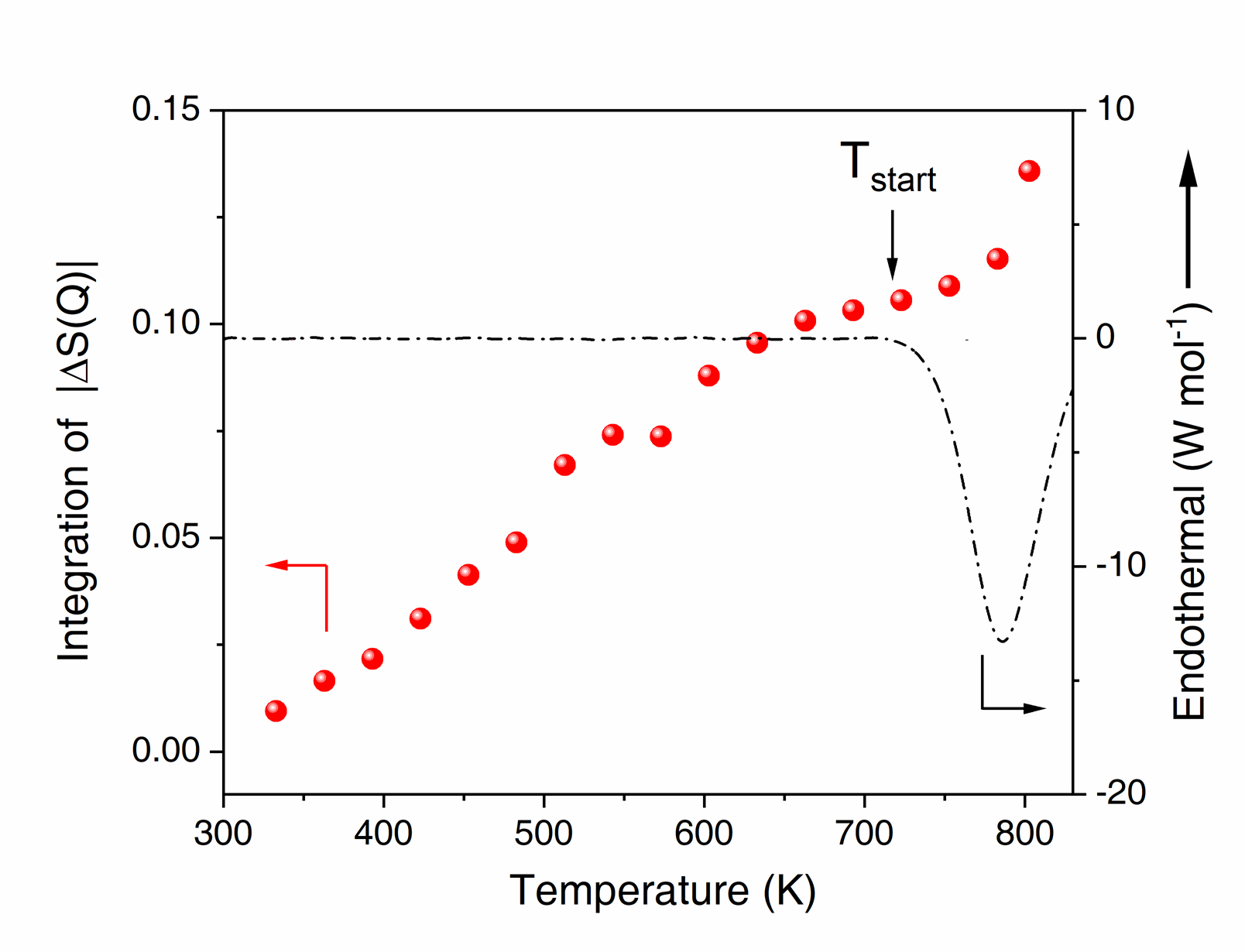
**

**Supplementary Figure 3. Integration of |*ΔS(Q)*| from 2 to 6 Å^-1^** **as a function of temperature.** The black dashed line is the DSC curve with a heating rate of 30 K min^-1^ for comparison.





**Supplementary Figure 4. The structural change of the NbNiZrTiCo HEMG sample as a function of temperature revealed by *in-situ* synchrotron XRD patterns and the corresponding DSC curve during heating.** **a** The first diffraction peak position (red circles) and the DSC curve (black dashed curve). **b** The first derivative of the principal diffraction peak position (red circles) during heating and the DSC curve (black dashed curve). The vertical dashed lines mark the *T*_start_ of the first exothermic peak in DSC, which agrees well with the change of the slope of the first diffraction peak position as marked by the two blue dashed lines in b. No obvious thermal lag is observed in the *in-situ* XRD experiment.

**
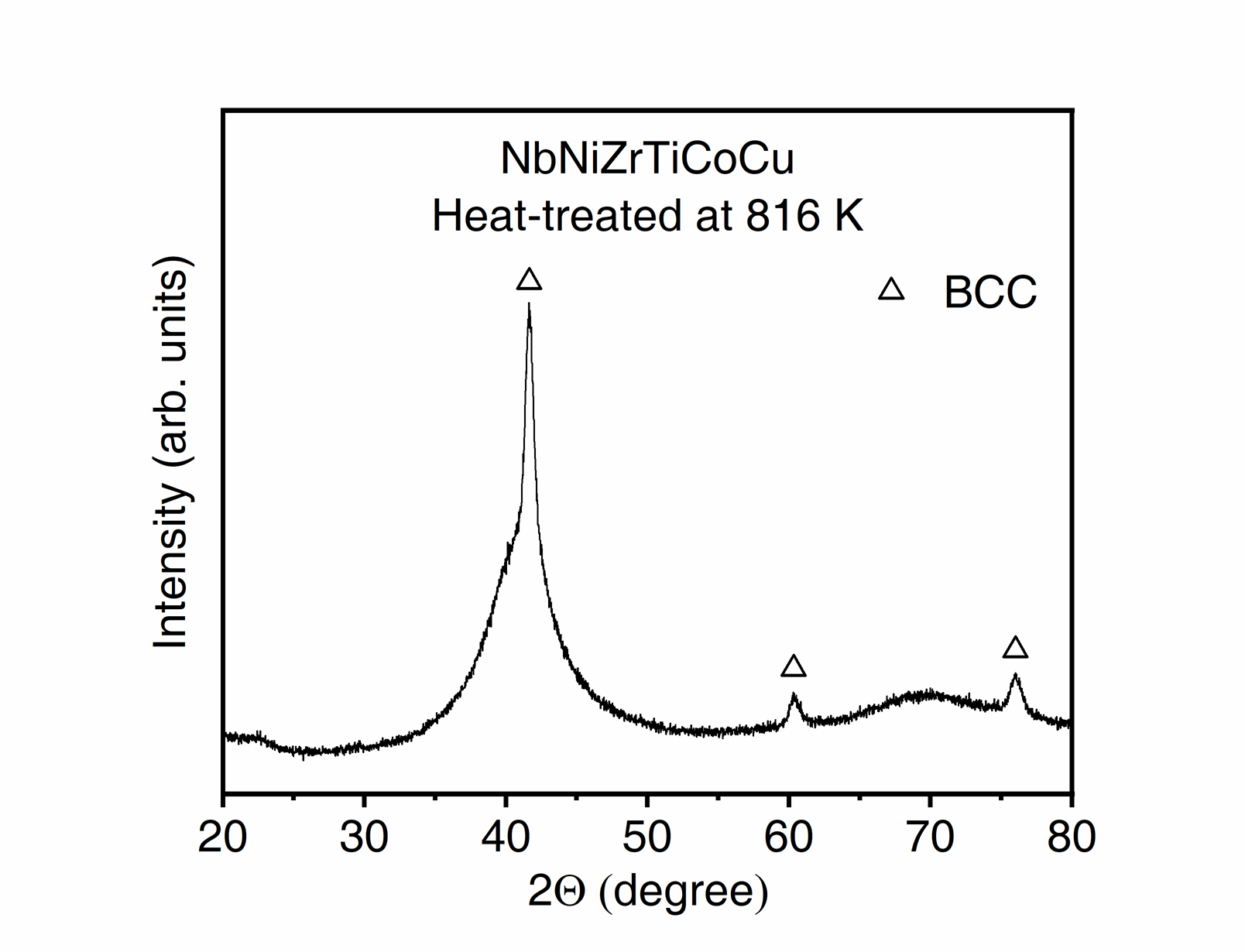
**

**Supplementary Figure 5. XRD results of the NbNiZrTiCoCu HEMG heated above the first exothermal peak (816 K).** The sample partially crystallizes and shows a dual-phase structure of body-centered cubic (BCC) phase and amorphous phase.


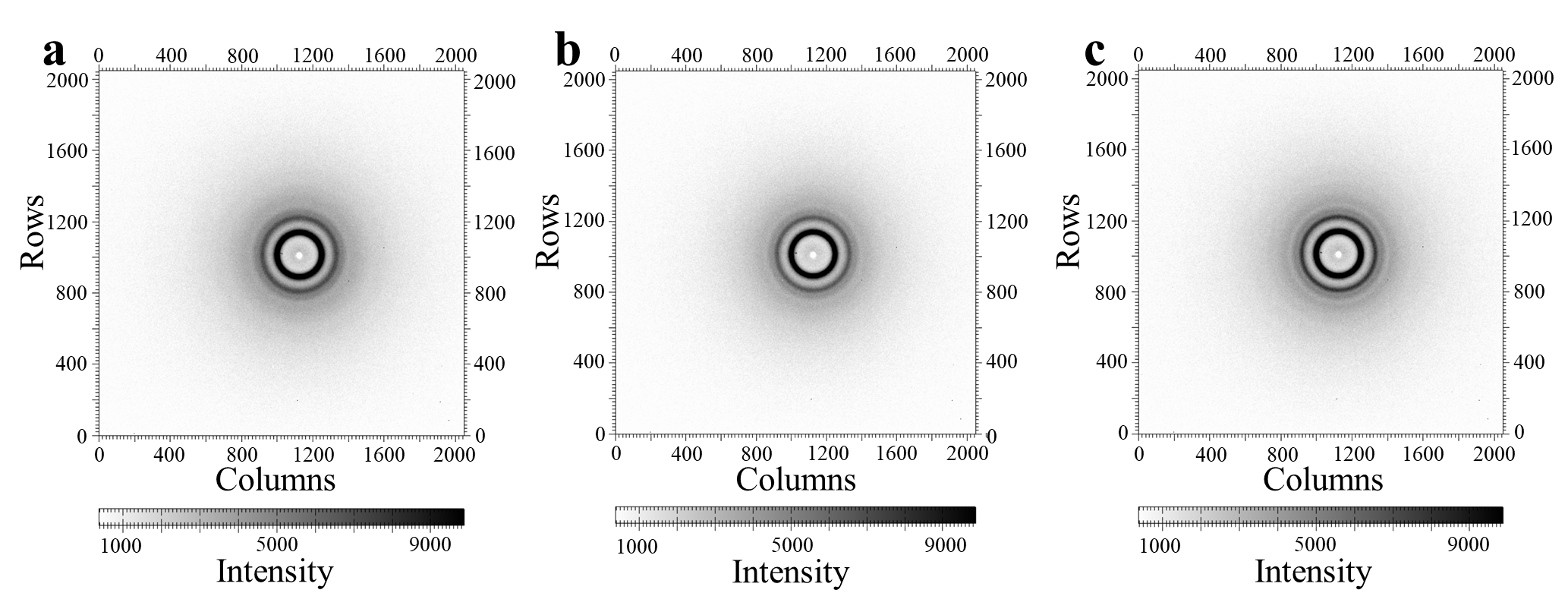


**Supplementary Figure 6. Two-dimensional *in-situ* synchrotron XRD images of the NbNiZrTiCo HEMG sample. a** The image at 303 K before heating. **b** The image at 803 K. **c** The image at 303 K after heating.

**Supplementary Tables**

**Supplementary Table 1. The *S*_C_, *S*_E_ and *S*_T_ of the HEMGs with glass-to-glass transitions, conventional Nb–Ni–Zr–based MGs, and HEMGs without glass-to-glass transitions.**

| **Category** | **Composition** | ***S*_C_ (J mol^-1^ K^-1^)** | ***S*_E_ (J mol^-1^ K^-1^)** | ***S*_T_ (J mol^-1^ K^-1^)** | **Ref.** |
| --- | --- | --- | --- | --- | --- |
| HEMGs with glass-to-glass transitions | Nb_20_Ni_20_Zr_20_Ti_20_Co_20_ | 13.38 | -2.75 | 10.63 | This work |
|  | Nb_20_Ni_20_Zr_20_Ti_20_Cu_20_ | 13.38 | -2.50 | 10.88 | This work |
|  | Nb_16.7_Ni_16.7_Zr_16.7_Ti_16.7_Co_16.7_Cu_16.7_ | 14.90 | -2.73 | 12.17 | This work |
| Nb–Ni–Zr–based MGs | Ni_62.25_Nb_32.75_Zr_5_ | 6.74 | -2.36 | 4.38 | 1 |
|  | Ni_62.25_Nb_30.75_Zr_5_Mo_2_ | 7.36 | -2.33 | 5.04 | 1 |
|  | Ni_62.25_Nb_30.75_Zr_5_V_2_ | 7.36 | -2.32 | 5.04 | 1 |
|  | Ni_62.25_Nb_30.75_Zr_5_Ta_2_ | 7.36 | -2.36 | 5.00 | 1 |
|  | Ni_62.25_Nb_28.75_Zr_5_Ta_4_ | 7.75 | -2.36 | 5.39 | 1 |
|  | Ni_62.25_Nb_30.75_Zr_5_Sn_2_ | 7.36 | -2.36 | 5.01 | 1 |
|  | Ni_62.25_Nb_30.75_Zr_5_Ti_2_ | 7.36 | -2.37 | 4.99 | 1 |
|  | Ni_62.25_Nb_28.75_Zr_5_Ti_4_ | 7.75 | -2.38 | 5.37 | 1 |
|  | Ni_60.25_Nb_32.75_Zr_5_Fe_2_ | 7.47 | -2.35 | 5.12 | 1 |
|  | Ni_58.25_Nb_32.75_Zr_5_Fe_4_ | 7.97 | -2.34 | 5.63 | 1 |
|  | Ni_60.25_Nb_32.75_Zr_5_Cu_2_ | 7.47 | -2.34 | 5.13 | 1 |
|  | Ni_58.25_Nb_32.75_Zr_5_Cu_4_ | 7.97 | -2.32 | 5.65 | 1 |
|  | Ni_60.25_Nb_32.75_Zr_5_Co_2_ | 7.47 | -2.36 | 5.12 | 1 |
|  | Ni_58.25_Nb_32.75_Zr_5_Co_4_ | 7.97 | -2.35 | 5.62 | 1 |
|  | (Ni_62.25_Nb_32.75_Zr_5_)_99_Si_1_ | 7.14 | -2.43 | 4.71 | 1 |
|  | (Ni_62.25_Nb_32.75_Zr_5_)_98_Sn_2_ | 7.42 | -2.35 | 5.07 | 1 |
|  | (Ni_62.25_Nb_32.75_Zr_5_)_98_Al_2_ | 7.42 | -2.34 | 5.08 | 1 |
| HEMGs without glass-to-glass transitions | Ti_20_Zr_20_Hf_20_Be_20_Cu_20_ | 13.38 | -4.59 | 8.79 | 2 |
|  | Ti_16.7_Zr_16.7_Hf_16.7_Be_16.7_Cu_16.7_Ni_16.7_ | 14.90 | -4.69 | 10.21 | 3 |
|  | Pd_20_Pt_20_Cu_20_Ni_20_P_20_ | 13.38 | -3.19 | 10.19 | 4 |
|  | Sr_20_Ca_20_Yb_20_Mg_20_Zn_20_ | 13.38 | -6.73 | 6.65 | 5 |
|  | Zr_31_Ti_27_Be_26_Cu_10_Fe_6_ | 12.19 | -5.04 | 7.14 | 6 |
|  | Zr_31_Ti_27_Be_26_Cu_10_Al_6_ | 12.19 | -4.79 | 7.39 | 6 |
|  | Zr_31_Ti_27_Be_26_Cu_10_Ag_6_ | 12.19 | -4.79 | 7.40 | 6 |
|  | Zr_31_Ti_27_Be_26_Cu_10_Ni_6_ | 12.19 | -5.10 | 7.09 | 6 |
|  | Zr_31_Ti_27_Be_26_Cu_10_Cr_6_ | 12.19 | -4.99 | 7.19 | 6 |
|  | Zr_31_Ti_27_Be_26_Cu_10_V_6_ | 12.19 | -4.88 | 7.31 | 6 |
|  | Zr_28_Ti_24_Be_23_Cu_9_Ni_10_Fe_6_ | 13.74 | -4.98 | 8.76 | 6 |
|  | Zr_28_Ti_24_Be_23_Cu_9_Ni_10_Al_6_ | 13.74 | -4.77 | 8.97 | 6 |
|  | Zr_28_Ti_24_Be_23_Cu_9_Ni_10_Ag_6_ | 13.74 | -4.77 | 8.97 | 6 |
|  | Zr_28_Ti_24_Be_23_Cu_9_Ni_10_Cr_6_ | 13.74 | -4.94 | 8.81 | 6 |
|  | Zr_28_Ti_24_Be_23_Cu_9_Ni_10_V_6_ | 13.74 | -4.83 | 8.91 | 6 |
|  | Fe_25_Co_25_Ni_25_P_10_C_7.5_B_5_Si_2.5_ | 14.19 | -5.04 | 9.15 | 7 |
|  | Fe_25_Co_25_Ni_25_P_10_C_5_B_5_Si_5_ | 14.29 | -4.14 | 10.16 | 7 |
|  | Fe_25_Co_25_Ni_25_P_12.5_C_2.5_B_5_Si_5_ | 14.06 | -3.37 | 10.69 | 7 |
|  | Fe_25_Co_25_Ni_25_P_7.5_C_5_B_7.5_Si_5_ | 14.37 | -4.47 | 9.90 | 7 |
|  | Fe_25_Co_25_Ni_25_P_10_C_2.5_B_7.5_Si_5_ | 14.19 | -3.70 | 10.49 | 7 |
|  | Fe_25_Co_25_Ni_25_P_10_C_2.5_B_5_Si_7.5_ | 14.19 | -3.23 | 10.95 | 7 |
|  | Ti_20_Zr_20_Hf_20_Be_20_Cu_17.5_Ni_2.5_ | 14.01 | -4.63 | 9.37 | 8 |
|  | Ti_20_Zr_20_Hf_20_Be_20_Cu_12.5_Ni_7.5_ | 14.48 | -4.73 | 9.75 | 8 |
|  | Ti_20_Zr_20_Hf_20_Be_20_Cu_7.5_Ni_12.5_ | 14.48 | -4.82 | 9.66 | 8 |
|  | Ti_20_Zr_20_Hf_20_Be_20_Cu_2.5_Ni_17.5_ | 14.01 | -4.92 | 9.09 | 8 |
|  | Fe_25_Co_25_Ni_25_B_15_Si_10_ | 12.92 | -3.65 | 9.27 | 9 |
|  | Fe_25_Co_25_Ni_25_B_17.5_Si_7.5_ | 12.80 | -4.12 | 8.68 | 9 |
|  | Ti_20_Zr_20_Hf_20_Be_20_Ni_20_ | 14.53 | -4.78 | 9.76 | 10 |
|  | La_30_Ce_30_Ni_10_Cu_10_Al_20_ | 12.51 | -5.97 | 6.54 | 11 |
|  | La_30_Ce_30_Ni_15_Cu_5_Al_20_ | 12.29 | -6.11 | 6.19 | 11 |
|  | La_35_Ce_25_Ni_10_Cu_10_Al_20_ | 12.44 | -6.04 | 6.40 | 11 |
|  | La_30_Ce_30_Ni_5_Cu_15_Al_20_ | 12.29 | -5.83 | 6.46 | 11 |
|  | La_25_Ce_35_Ni_10_Cu_10_Al_20_ | 12.44 | -5.90 | 6.54 | 11 |
|  | Gd_20_Tb_20_Dy_20_Co_20_Al_20_ | 13.38 | -5.11 | 8.27 | 12 |
|  | Gd_20_Tb_20_Dy_20_Ni_20_Al_20_ | 13.38 | -5.25 | 8.14 | 12 |
|  | Gd_20_Tb_20_Dy_20_Fe_20_Al_20_ | 13.38 | -4.98 | 8.41 | 12 |
|  | Ho_16.5_Er_38.5_Al_27.5_Co_17.5_ | 11.01 | -4.58 | 6.43 | 13 |
|  | Er_20_Dy_20_Co_20_Al_20_Gd_20_ | 13.38 | -5.05 | 8.33 | 14 |
|  | Er_20_Dy_20_Co_20_Al_20_Tb_20_ | 13.38 | -4.87 | 8.51 | 14 |
|  | Er_20_Dy_20_Co_20_Al_20_Tm_20_ | 13.38 | -4.80 | 8.58 | 14 |
|  | Gd_25_Co_25_Al_25_Y_25_ | 11.53 | -6.13 | 5.40 | 15 |
|  | Gd_25_Co_25_Al_25_Y_15_Dy_10_ | 12.92 | -6.06 | 6.87 | 15 |
|  | Gd_25_Co_25_Al_25_Y_15_Ho_10_ | 12.92 | -6.00 | 6.93 | 15 |
|  | Gd_25_Co_25_Al_25_Y_15_Er_10_ | 12.92 | -6.00 | 6.93 | 15 |
|  | Gd_25_Co_25_Al_25_Ho_24_Y_1_ | 11.87 | -5.81 | 6.07 | 16 |
|  | Gd_25_Co_25_Al_25_Ho_20_Y_5_ | 12.57 | -5.86 | 6.70 | 16 |
|  | Gd_25_Co_25_Al_25_Ho_15_Y_10_ | 12.92 | -5.93 | 6.99 | 16 |
|  | Gd_25_Co_25_Al_25_Ho_10_Y_15_ | 12.92 | -6.00 | 6.93 | 16 |
|  | Gd_25_Tb_25_Co_25_Al_25_ | 11.53 | -5.88 | 5.64 | 17 |
|  | Gd_25_Dy_25_Co_25_Al_25_ | 11.53 | -5.95 | 5.57 | 17 |
|  | Gd_25_Ho_25_Co_25_Al_25_ | 11.53 | -5.79 | 5.73 | 17 |
|  | Fe_25_Co_25_Ni_25_Mo_5_P_10_B_10_ | 13.72 | -3.48 | 10.23 | 18 |

**Supplementary Notes**

**Supplementary Note 1. Flash DSC curves.**

For the as-prepared NbNiZrTiCo HEMG, its *T*_g_ cannot be detected by using the conventional DSC measurement (Supplementary Fig. **1a** in the manuscript). However, using a chip-based Flash DSC with a much faster heating rate of 500-8000 K s^-1^ (Supplementary Fig. **7a**), the *T*_g_ of the as-prepared NbNiZrTiCo HEMG can be clearly observed before the first exothermic peak with heating rates higher than 1000 K s^-1^. According to these results, it can be claimed that the phase transition is a liquid-to-liquid transition when the heating rate is higher than 1000 K s^-1^.

We have also tried to estimate the *T*_g_ in the DSC curve with a regular heating rate of 0.333 K s^-1^ (20 K min^-1^) by extrapolation (Supplementary Fig. **7b**). According to the Kissinger equation fitting (same function as in Supplementary Note 2) of the flash DSC data with various heating rates, the *T*_g_ is estimated to be 629 K with a regular heating rate of 0.333 K s^-1^. However, the estimated *T*_g_ is not observed in the experimental result, and actually no significant structural change is observed before the transition. From this aspect, it may not be solid to claim that the discovered transition in the manuscript is a liquid-to-liquid transition at a heating rate of 0.333 K s^-1^ due to the absence of *T*_g_. However, since the heat-treated sample also shows a *T*_g_ of 764 K (Fig. **1b** in the manuscript), the as-prepared sample and the heat-treated sample are both in glass states and thus the “glass-to-glass transition” can be well confirmed. We do not deny that such a glass-to-glass transition could be a potential liquid-to-liquid transition with a regular heating rate, but to avoid possible dispute or confusion, it may be better to use the term “glass-to-glass transition” instead of “liquid-to-liquid transition” to describe the experimental results.

**
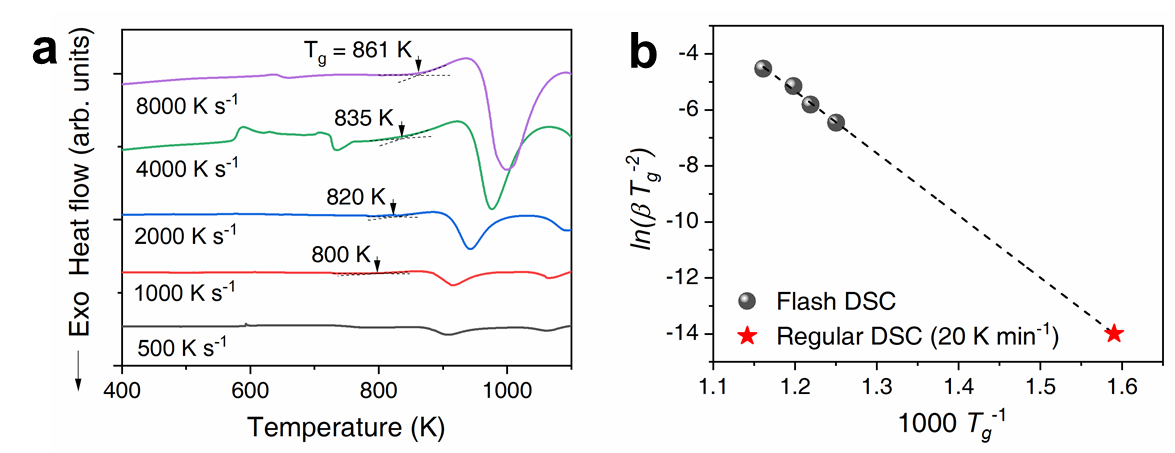
**

**Supplementary Figure 7. Flash DSC results and the estimation of the *T_g_* with a regular heating rate. a** Flash DSC curves with different heating rates. **b** Fitting of the *T_g_* with different heating rates using the Kissinger equation. The stars are extrapolated data at a conventional heating rate of 0.333 K s^-1^ (20 K min^-1^).

**Experimental procedure:** The Flash DSC measurement was performed using a Mettler-Toledo Flash DSC 2+ with a UFH 1 sensor. The initial ribbon sample was cut into small pieces of 40 μm * 40 μm under a stereomicroscope and then loaded on the Flash DSC chip. The sample support temperature of the Flash DSC was set to 183 K (−90 °C), and the furnace was purged with argon at a flow rate of 80 ml min^-1^. The samples were heated with a series of heating rates from 500 K s^-1^ to 8000 K s^-1^.

**Supplementary Note 2. DSC curves with multiple heating rates.**

DSC analyses with multiple heating rates are performed (10, 20, 30, 40, and 50 K min^-1^, covering the heating rate of the *in-situ* diffraction, Supplementary Fig. **8a**), and the activation energies corresponding to the three exothermic peaks are obtained (Supplementary Fig. **8b**) by fitting the DSC results with the Kissinger function as below

$\ln(\frac{\beta}{T_{p}^{2}})=-\frac{E}{R}\frac{1}{T_{p}}+C$ (1)

where $\beta$ is the heating rate, $T_{p}$is the peak exothermic temperature, $R$ is the ideal gas constant, $E$ is the activation energy, and $C$ is a constant. The Kissinger function fits the experimental data well (Supplementary Fig. **8b**), and the three exothermic peaks show activation energies of 281, 384, and 249 kJ mol^-1^, respectively.

**
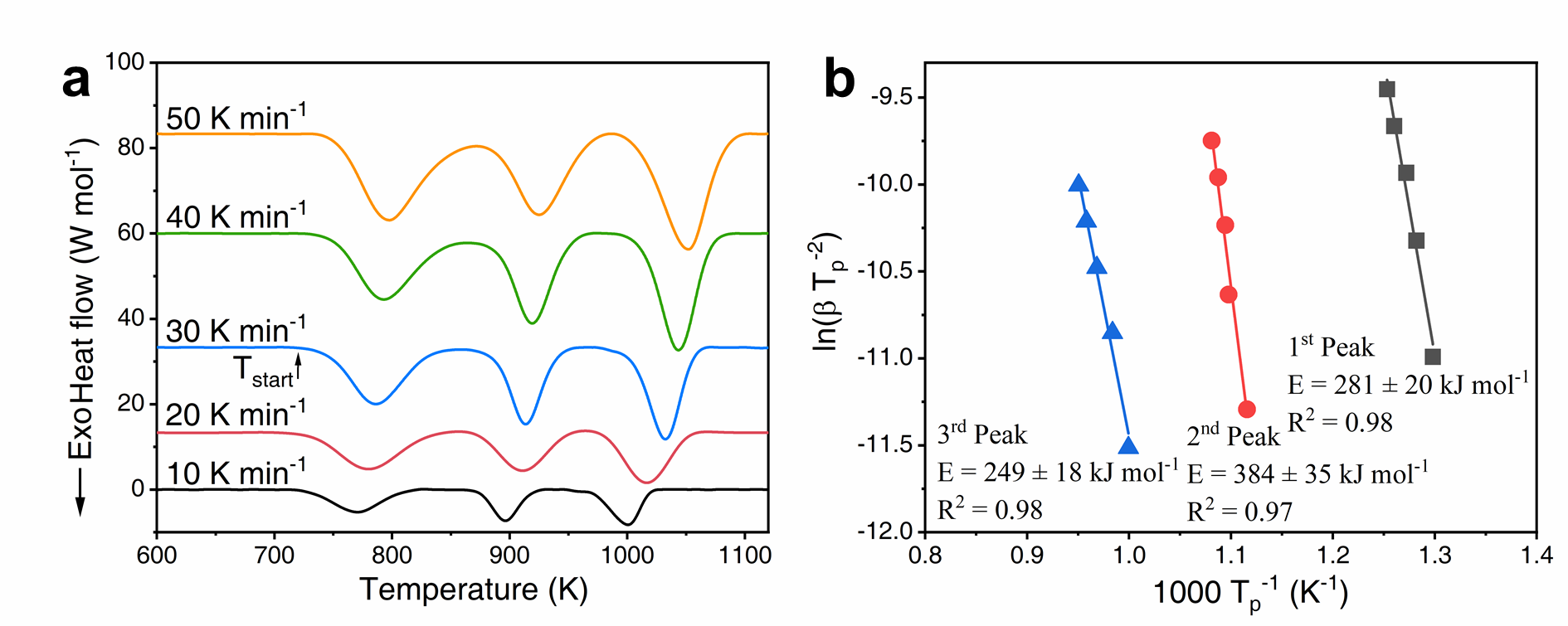
**

**Supplementary Figure 8. DSC curves with multiple heating rates. a** DSC curves of NbNiZrTiCo HEMG with heating rates of 10, 20, 30, 40, and 50 K min^-1^. The start temperature of the glass-to-glass transition with a heating rate of 30 K min^-1^ is denoted as *T*_start_ (717 K). **b** Kissinger plots corresponding to the three exothermic peaks.

**Supplementary Note 3. Additional HRTEM images.**

To confirm the observed microstructures, supplementary HRTEM measurements are conducted on multiple spots of each heat-treated sample with the three different compositions (Supplementary Fig. **9**: NbNiZrTiCo; Supplementary Fig. **10**: NbNiZrTiCu; Supplementary Fig. **11**: NbNiZrTiCoCu), and the results show that none of the heat-treated samples presents any feature of nano-crystallization in the HRTEM images or SAED patterns. The fully crystallized microstructure of the NbNiZrTiCo HEMG heated to *T*_B_ is also confirmed by the HRTEM results (Supplementary Fig. **12**).


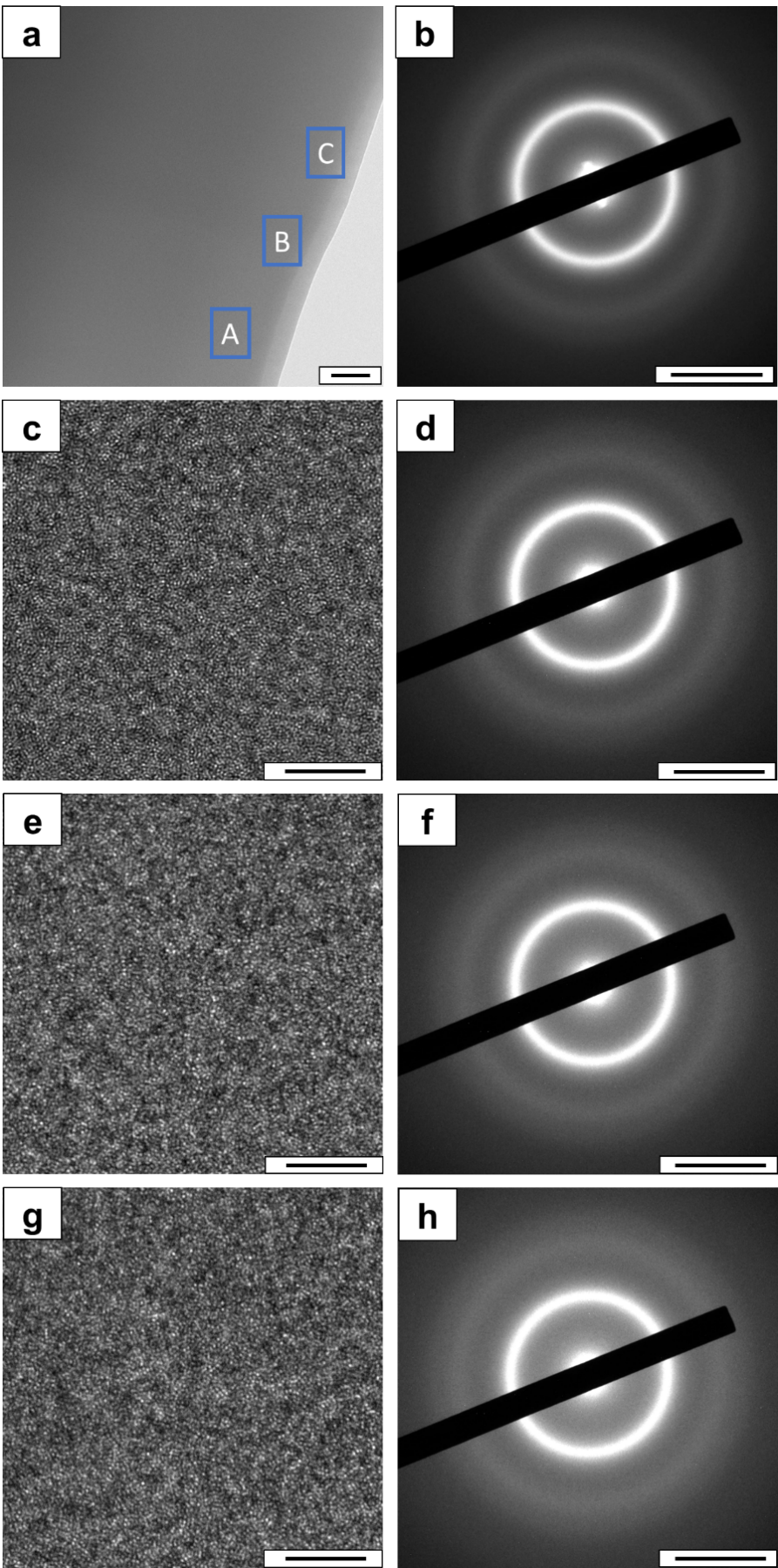


**Supplementary Figure 9. Additional TEM and SAED results of the heat-treated NbNiZrTiCo sample.** **a** Bright-field TEM image of the heat-treated sample (scale bar: 200 nm). **b** SAED image (scale bar: 5 nm^-1^) of the heat-treated sample. **c** HRTEM (scale bar: 5 nm) image of the A area in a. **d** SAED image (scale bar: 5 nm^-1^) of the A area in **a**. **e, f** Same as **c, d** but for the B area. **g, h** Same as **c, d** but for the C area.


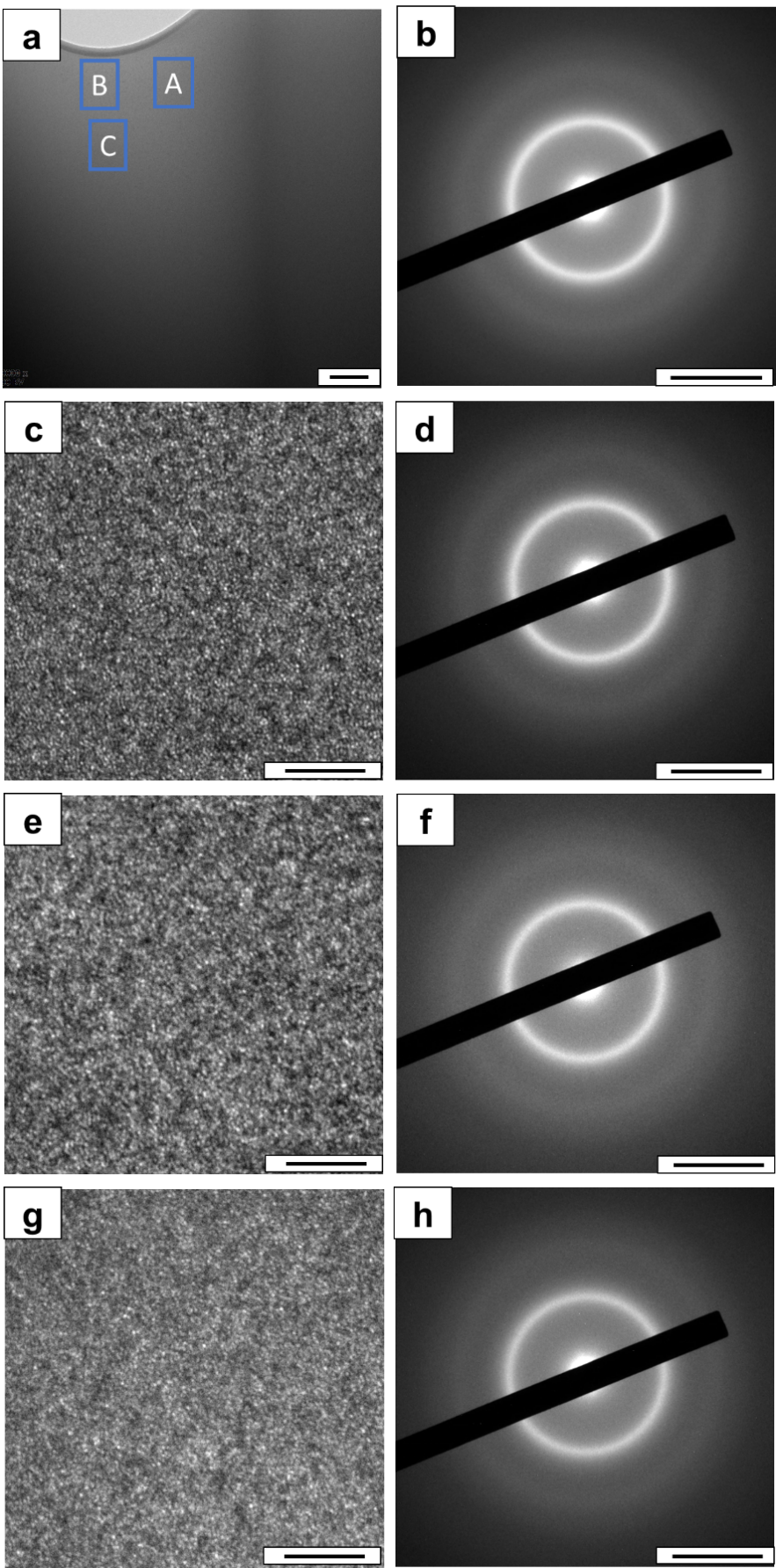


**Supplementary Figure 10. Additional TEM and SAED results of the heat-treated NbNiZrTiCu sample.** **a** Bright-field TEM image of the heat-treated sample (scale bar: 200 nm). **b** SAED image (scale bar: 5 nm^-1^) of the heat-treated sample. **c** HRTEM (scale bar: 5 nm) image of the A area in a. **d** SAED image (scale bar: 5 nm^-1^) of the A area in **a**. **e, f** Same as **c, d** but for the B area. **g, h** Same as **c, d** but for the C area.


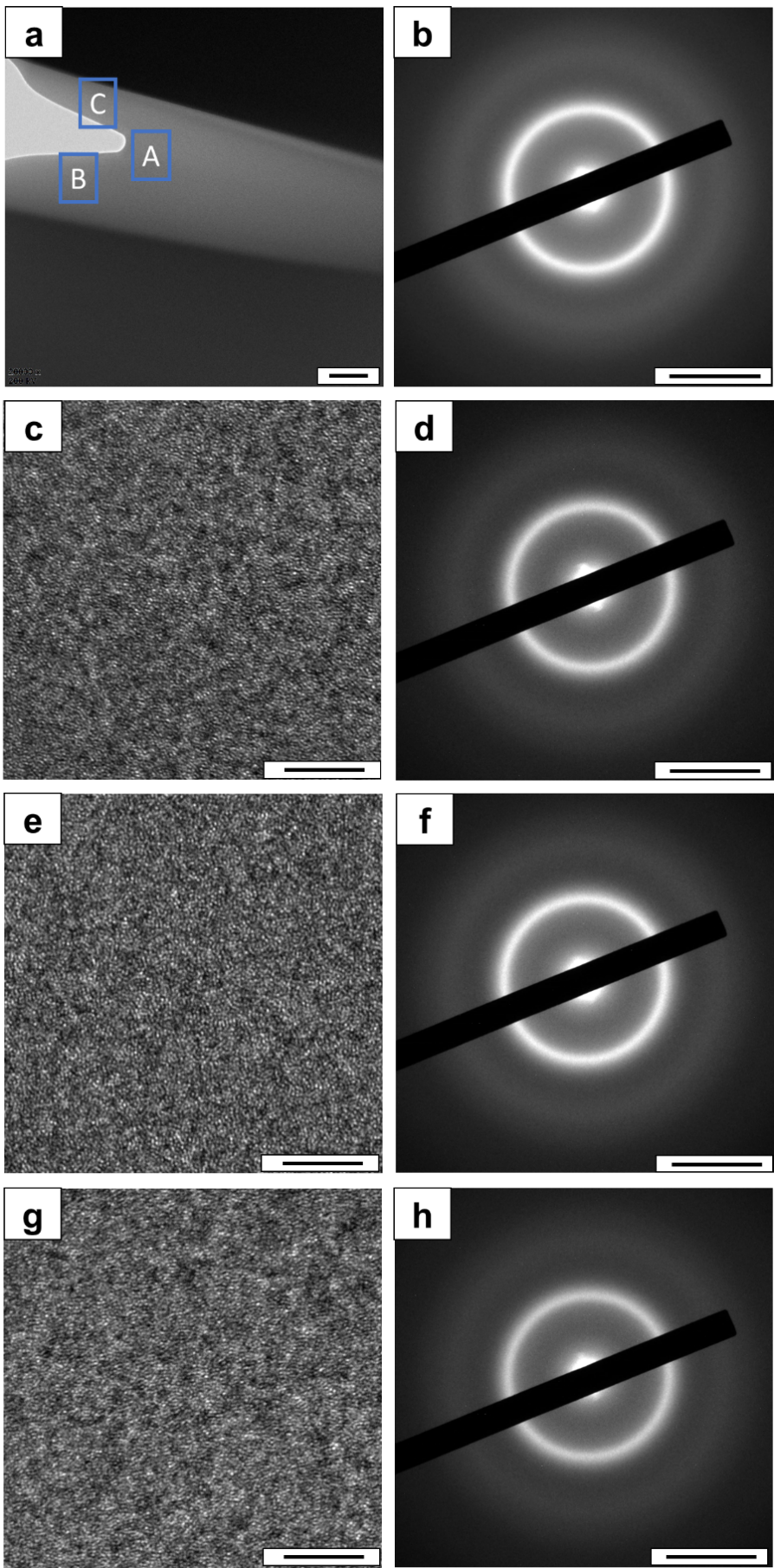


**Supplementary Figure 11. Additional TEM and SAED results of the heat-treated NbNiZrTiCoCu sample.** **a** Bright-field TEM image of the heat-treated sample (scale bar: 200 nm). **b** SAED image (scale bar: 5 nm^-1^) of the heat-treated sample. **c** HRTEM (scale bar: 5 nm) image of the A area in a. **d** SAED image (scale bar: 5 nm^-1^) of the A area in **a**. **e, f** Same as **c, d** but for the B area. **g, h** Same as **c, d** but for the C area.


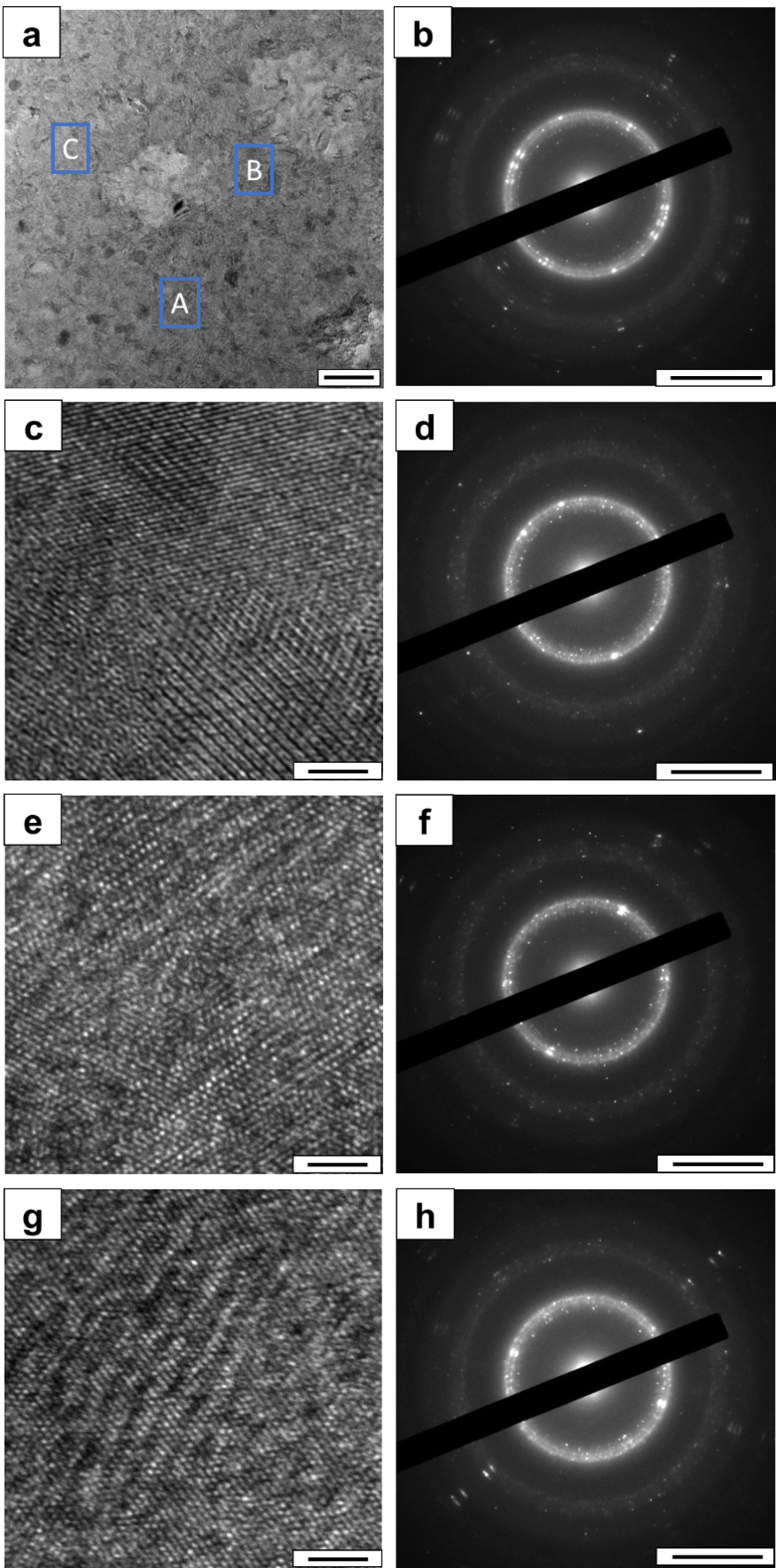


**Supplementary Figure 12. Additional TEM and SAED results of the NbNiZrTiCo sample heat-treated at *T*_B_.** **a** Bright-field TEM image of the sample (scale bar: 100 nm). **b** SAED image (scale bar: 5 nm^-1^) of the sample. **c** HRTEM (scale bar: 2 nm) image of the A area in a. **d** SAED image (scale bar: 5 nm^-1^) of the A area in **a**. **e, f** Same as **c, d** but for the B area. **g, h** Same as **c, d** but for the C area.

**Experimental procedure:** The TEM thin foils were extracted from the ribbons by the Zeiss Auriga focused ion beam (FIB) system with Ga^+^ ion beams. The Ar^+^ ion beams were used for further thinning and polishing by the Model 1040 NanoMill® TEM specimen preparation system, with an energy of 900 eV and a current of 150 μA. The bright-field TEM images, HRTEM images and the diffraction images were attained by the JEOL JEM-2100F transmission electron microscope with a LaB_6_ electron source operated at 200 kV.

**Supplementary Note 4. Synchrotron small-angle X-ray scattering results.**

To characterize the structural and compositional homogeneity at the macro-scale, synchrotron small-angle X-ray scattering (SAXS) experiments were performed on the as-prepared and heated-treated NbNiZrTiCo samples with an X-ray beam size of ~0.5*0.5 mm^2^. Both SAXS curves (Supplementary Fig. **13**) are smoothly decayed as a function of the wave vector, *Q*, with no detectable interference peak over the entire *Q* range, suggesting that there is no obvious density fluctuation typically associated with composition segregation or density change due to crystallization at the length scale over 1 nm, which is consistent with the APT and HRTEM analysis. The small glitch at *Q* = 0.06 Å^-1^ is caused by the gaps in the two-dimensional Pilatus detector. The overall intensity difference between the two samples is supposed to be caused by the different sample thicknesses and structures.





**Supplementary Figure 13. Synchrotron SAXS results for the as-prepared and heated-treated NbNiZrTiCo samples.** Both SAXS curves are smooth with no detectable interference peak over the entire *Q* range, suggesting no obvious density fluctuation at the length scale over 1 nm. The small glitch at *Q* = 0.06 Å^-1^ is caused by the gaps in the two-dimensional Pilatus detector. The overall intensity difference between the two samples is supposed to be caused by different sample thicknesses and differences in their atomic structures.

**Experimental procedure:** Small-angle X-ray scattering (SAXS) experiments were performed at the beamline BL16B1 of the Shanghai Synchrotron Radiation Facility (SSRF). The wavelength of the X-ray beam was 1.0332 Å (12.0 keV). The beam size was 0.5 mm × 0.5 mm. SAXS was performed in a transmission mode by using a 2D Pilatus 2M 3S detector and a pixel size of 172*172 μm^2^. The two samples were prepared using the same protocol as the synchrotron X-ray diffraction experiments.

**Supplementary Note 5. Thermal stability of the NbNiZrTiCo HEMG.**

Isothermal DSC analyses are performed to reveal the time dependence of the stability of the HEMG. It is found that the heat-treated sample remains amorphous after annealed at *T*_g_ - 5 K (759 K) for 2 hours, *T*_g_ + 30 K (794 K) for 2 hours, and *T*_g_ + 30 K (794 K) for 10 hours. To avoid the severe surface oxidation of the samples, the upper limit of the time periods for the isothermal scans is selected to be 10 hours. We found that the heat-treated sample remains amorphous even after annealed at *T*_g_ + 30 K (794 K) for 10 hours. Therefore, below *T*_g_, we only selected the temperature of *T*_g_ - 5 K (759 K) for the isothermal scan analyses. During the annealing at *T*_g_ - 5 K (759 K) for 2 hours, *T*_g_ + 30 K (794 K) for 2 hours and *T*_g_ + 30 K (794 K) for 10 hours, no exothermic peak is observed in any of the isothermal scan curves (Supplementary Figs. **14a**, **d** and **g**), indicating no crystallization during the annealing and a high crystallization resistance. The XRD patterns of the annealed samples show no sharp Bragg peaks (Supplementary Figs. **14b**, **e** and **h**) and the latter two exothermic peaks still can be observed in the DSC curves (Supplementary Figs. **14c**, **f** and **i**). These results show that the HEMG possesses excellent thermal stability, and indicate that the glass-to-glass transition enable us to realize an ultrastable low-energy glass state, whose low energy makes it more difficult to crystallize than the conventional metallic glasses.


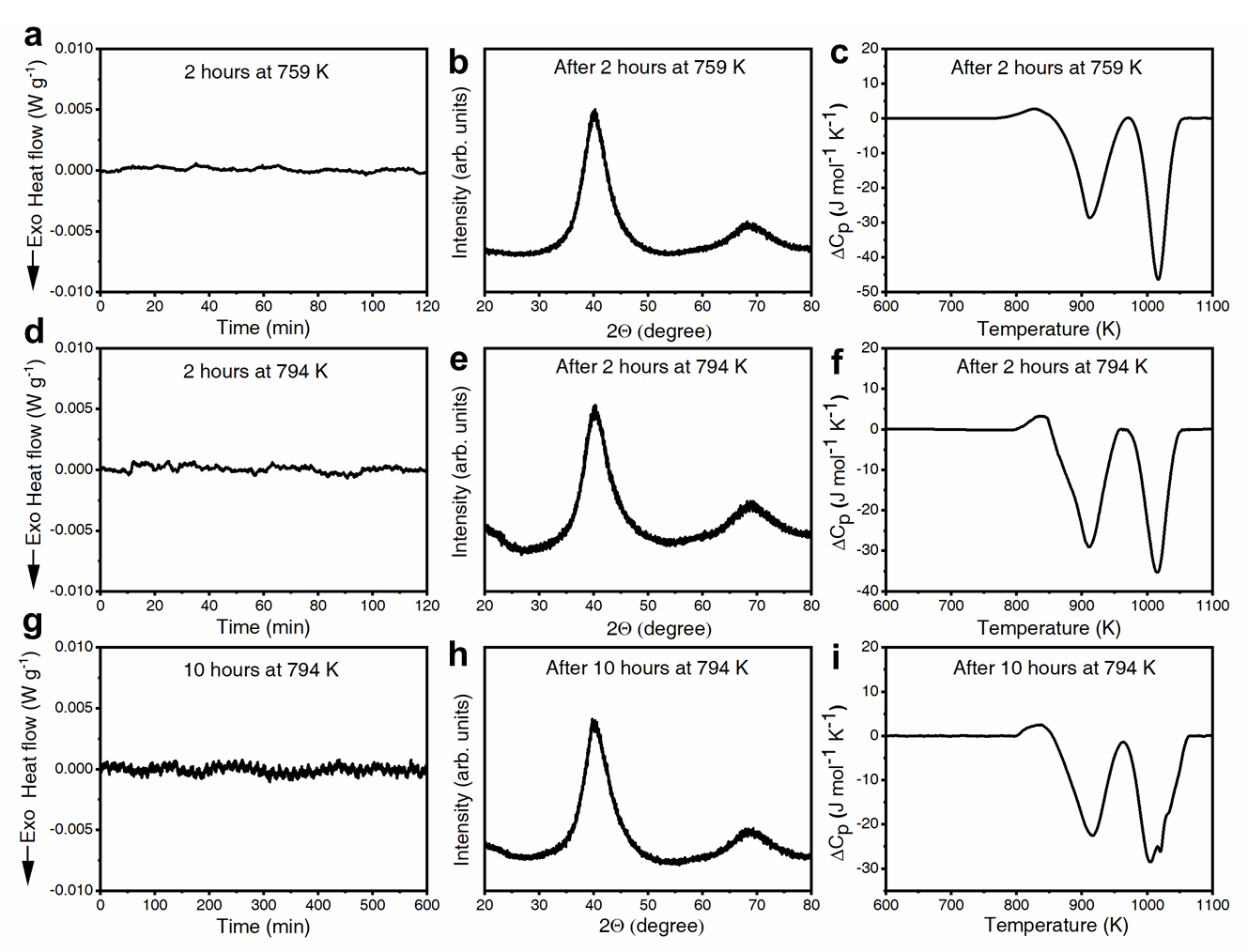


**Supplementary Figure 14. Isothermal DSC curves, X-ray diffraction analyses and speciﬁc heat (*ΔC*_P_) curves of the annealed samples. a** Isothermal DSC curves of annealing at *T*_g_ - 5 K (759 K) for 2 hours. **b** XRD curve of the sample annealed at *T*_g_ - 5 K (759 K) for 2 hours. **c** DSC curve of the sample annealed at *T*_g_ - 5 K (759 K) for 2 hours. (d-f) Same as (a-c) but annealed at *T*_g_ + 30 K (794 K) for 2 hours. (g-i) Same as (a-c) but annealed at *T*_g_ + 30 K (794 K) for 10 hours. No obvious exothermic peak is observed in the isothermal DSC curves. All annealed samples remain amorphous and the latter two exothermic peaks can be observed in the DSC curves.

**Experiment procedure:** DSC curves were measured using Al_2_O_3_ crucibles with a synchronous thermal analyzer (STA-449 F3, NETZSCH, Germany) under a high-purity argon atmosphere. The weight of each tested sample was ~20 mg. For multiple heating rates experiments, the heating rates were 10, 20, 30, 40, and 50 K min^-1^. For isothermal scans experiments, the heat-treated samples were heated to *T_g_* - 5 K (759 K) or *T_g_* + 30 K (794 K) with a heating rate of 20 K min^-1^ and kept for 2 or 10 hours. Then, the samples were cooled down to room temperature and followed by a DSC scan with a heating rate of 20 K min^-1^. To highlight the phase transitions, the corresponding DSC curve of the crystallized HEMG (samples heated to 1173 K) was subtracted. The XRD analyses were performed using an X-ray diffractometer (D/max-RB, Rigaku Inc., Japan) with Cu *Kα* radiation (wavelength 1.5406 Å) at room temperature. The XRD experiments were performed using a *θ-2θ* mode with a scanning rate of 3 degrees min^-1^.

**Supplementary Note 6. Additional APT measurements and quantitative analyses**

To unambiguously verify the APT results, the APT measurements are repeated on additional samples (Supplementary Fig. **15**), and the results confirm the homogeneous elemental distributions without any segregation. Moreover, a quantitative analysis of the four sets of APT data is carried out (Supplementary Fig. **16**). Specifically, to show the randomness of the atom distribution quantitatively, the experimental frequency distribution curves of each element are obtained by counting the number of atoms of a given element n_A_ in the sampling volumes containing a fixed total number of *N* atoms (Supplementary Fig. **16**). If the element A is randomly distributed in the sample, the frequency distribution *e*(*n*_A_) is theoretically expected to follow the binomial distribution *B*(*n*_A_) as below^19^:

$e(n_{A})=N\cdot B(n_{A})=N\cdot\frac{N!}{n_{A}!(N-n_{A})!}c_{A}^{n_{A}}(1-c_{A})^{N-n_{A}}$ (2),

where *c*_A_ is the mole fraction of the element A. The experimental frequency distribution curves fit well with the binomial distribution, as shown in Supplementary Fig. **16**. The statistical distance between the observed and expected distribution is evaluated by the reduced *χ*^2^ as^19^

$\chi^{2}=\sum_{n_{A}=0}^{N} \frac{(e(n_{A})-O(n_{A}))^{2}}{e(n_{A})}$ (3),

where *O*(*n_A_*) is the observed frequency distribution. A smaller reduced *χ*^2^ indicates that the observed frequency distribution is closer to the binomial distribution. However, the reduced *χ*^2^ is dependent on the sample size. To effectively normalize the dependence of sample size, the normalized homogenization parameter *μ* is calculated as^20^:

$\mu=\sqrt{\frac{\chi^{2}}{N+\chi^{2}}}$ (4).

The *μ* is independent of the sample size, and its value is between 0 and 1, where 0 indicates an ideally random distribution, and 1 indicates a complete association in the occurrence of the A atoms^20^. The calculated *μ* parameters are listed in Supplementary Table **2**, where the *μ* for all elements are small and close to 0, quantitatively confirming the random spatial distribution of all the elements in both the as-prepared and heat-treated alloys.


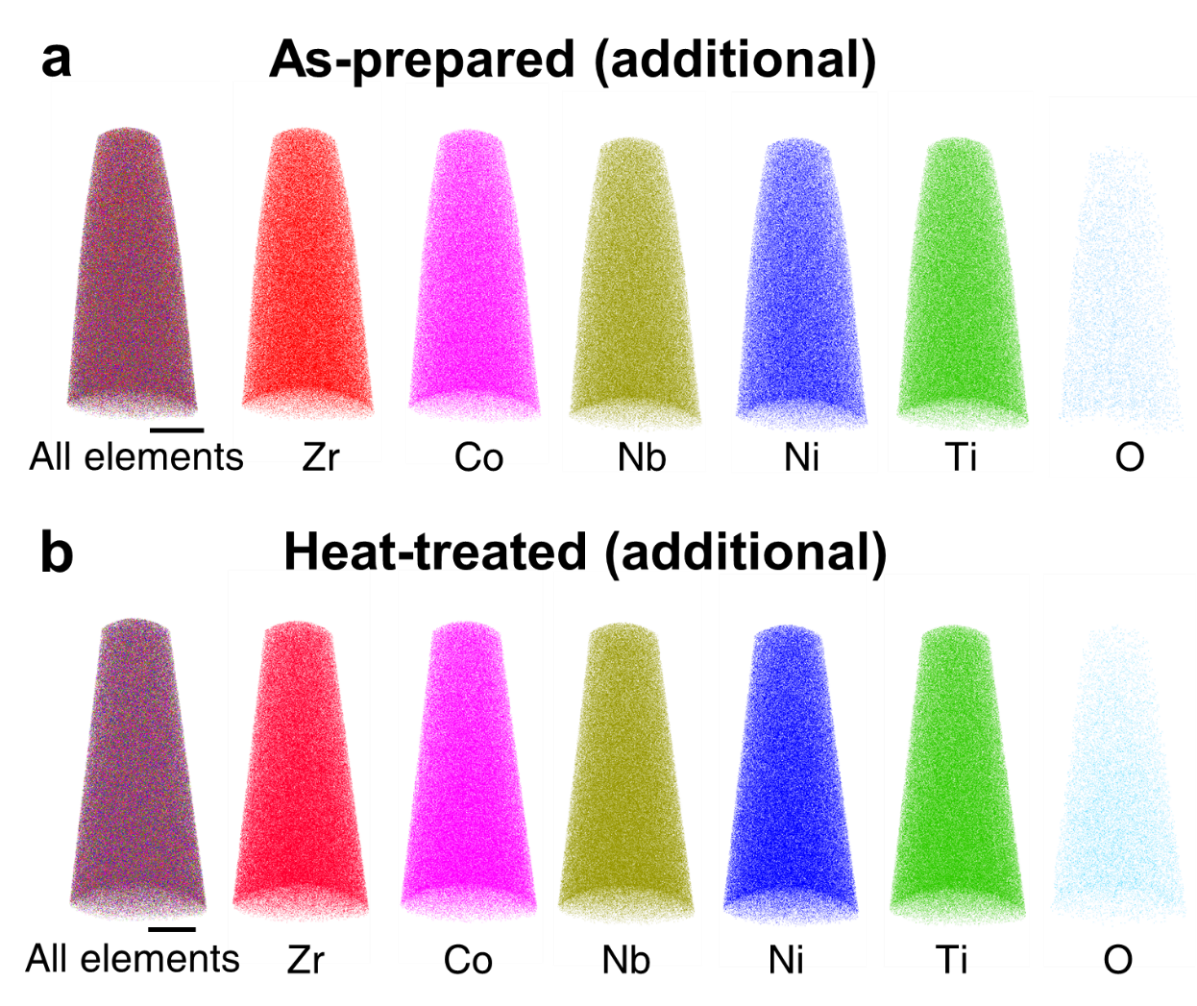


**Supplementary Figure 15. 3D APT reconstruction of the additional as-prepared (a) and heat-treated (b) NbNiZrTiCo samples.** The results show no compositional heterogeneity as well. The scale bars represent 20 nm.


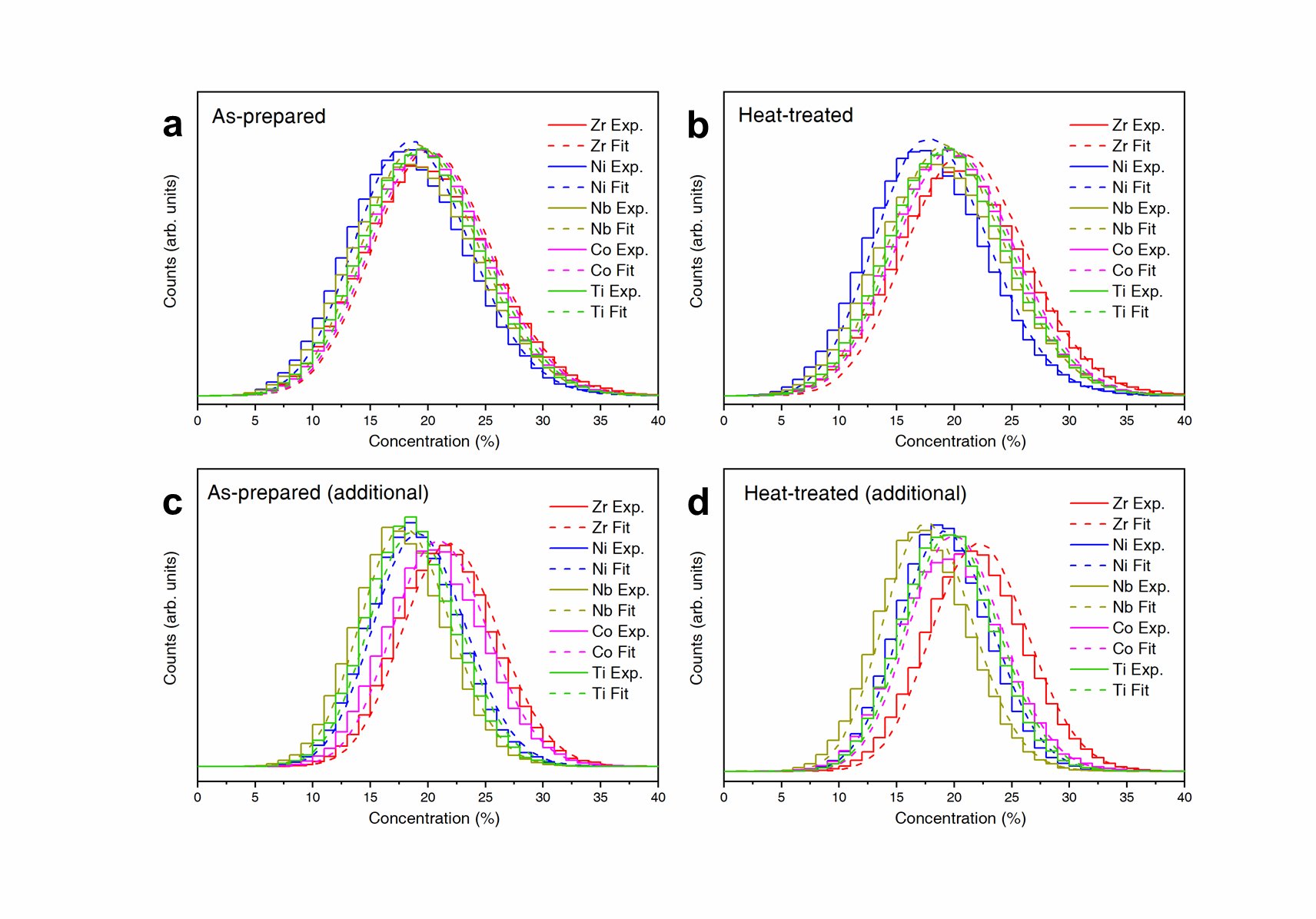


**Supplementary Figure 16. The frequency distribution curves and the fitted binomial distribution curves of each element distribution by analyzing the APT data of the as-prepared (a, c) and heat-treated (b, d) NbNiZrTiCo samples.** Experimental results are shown in solid curves while fitting results are shown in dashed curves.

**Supplementary Table 2. Normalized homogenization parameter *μ* of the frequency distribution curves.**

| **Sample** | **Element** | ***μ*** |
| --- | --- | --- |
| as-prepared | Nb | 0.1331 |
|  | Ni | 0.0918 |
|  | Zr | 0.1100 |
|  | Ti | 0.0451 |
|  | Co | 0.0330 |
| heat-treated | Nb | 0.1695 |
|  | Ni | 0.0877 |
|  | Zr | 0.1156 |
|  | Ti | 0.0417 |
|  | Co | 0.0431 |
| additional as-prepared | Nb | 0.0364 |
|  | Ni | 0.0361 |
|  | Zr | 0.0487 |
|  | Ti | 0.0480 |
|  | Co | 0.0881 |
| additional heat-treated | Nb | 0.1365 |
|  | Ni | 0.0563 |
|  | Zr | 0.0714 |
|  | Ti | 0.0299 |
|  | Co | 0.0293 |

**Supplementary Note 7. Fitting of the first peak in *G(r)***

There are 15 different partial pair distribution functions in the NbNiZrTiCo metallic glass. However, it is so far technically impossible to separate the *G(r)* curve into 15 partial pair distribution functions due to their severe overlapping. Instead, we mainly focus on the shape changes of the first two sub-peaks of *G(r)*, which conveniently reflects the local structural evolution involving the short-range order. To quantitatively evaluate the shape changes of the peaks, we attempted to use minimum numbers of Gaussian functions to fit them and found that the first peak in *G(r)* can be well fitted by two Gaussian functions as shown in Supplementary Fig. **17a**. Although the two Gaussian peaks cannot be directly assigned to any specific atomic pairs due to the uncertainty in their positions and weights, the analysis on the temperature dependence of the first peak shape in *G(r)* fitted by two Gaussian peaks still helps to quantitatively describe the short-range structural evolution as a function of temperature. For example, these analyses give quite consistent results showing a transition at around *T_start_* temperature (Fig. **4** in the main text). Similar peak fitting methods for the first peak in *G(r)* have been extensively used in the metallic glass community to derive structural information of multicomponent systems quantitatively^21-23^.

To derive more information of the short-range structure, we qualitatively compared the atomic pair distances and their partial weights with the radial distribution functions (RDFs) of the as-prepared sample and the sample cooled down from 803 K in the *in-situ* experiment (Supplementary Fig. **17b**). For a completely random quinary system, the fraction of a specific homoatomic pair is$\frac{1}{5}*\frac{1}{5}\text{=0.04}$, and the fraction of a specific heteroatomic pair is$\frac{1}{5}*\frac{1}{5}*\text{2=0.08}$. Given the X-ray scattering weight of each atomic pair, the sum-weight of each atomic pair is

,

where the $w_{ij}$ is the X-ray scattering weight. The 15 atomic pairs are grouped into six groups due to the similar bond lengths of some atomic pairs, and the sum-weights of the atomic pairs in the same group are added for the convenience of comparison.

It can be observed that the as-prepared sample resembles the random distribution with possibly maximized configurational entropy, while the sample cooled down from 803 K to room temperature considerably deviate from the initial random distribution with enhanced intensity in the atomic pair distance between 2.8 and 3.2 Å.

In the NbNiZrTiCo HEMG, the Zr/Ti elements, as well as the Co/Ni elements have similar chemical properties and zero heat of mixing, while other atomic pairs show positive or negative heats of mixing as shown in Supplementary Fig. **18a**. Therefore, for simplicity in our illustration, Zr and Ti (Co and Ni) are considered as similar atoms in the schematic image. As shown in Supplementary Fig. **18b**, the as-prepared MG was quenched into a highly disordered state from the melt, leading to a more random distribution of different elements mainly dominated by the high-entropy effect. During heating, the structural ordering via atomic rearrangement occurs mainly in the short-range. The Ti/Zr elements have positive mixing enthalpy with the Nb element, and the Co/Ni elements have more negative mixing enthalpy with the Zr/Ti elements than that between the Co/Ni elements and the Nb element. As a result, more short-range ordered clusters are formed among those elements with more negative heats of mixing (Supplementary Fig. **18c**). In contrast, the atoms surrounding these closely packed atomic clusters are more loosely distributed due to the positive or less negative heats of mixing, which is consistent with the analysis of the fitting of the first peak of *G(r)* and the rise of the “second sub-peak” in the first peak in radial distribution functions shown in Supplementary Fig. **17**. Eventually, these enthalpy-driven atomic rearrangements result in a more ordered distribution of the atoms, yielding a smaller full width at half maximum (FWHM) of the first diffraction peak of the *S*(*Q*) (Fig. **3d** in the manuscript). Besides, we would like to point out that much less is known about the detailed structures of the metallic glasses^24^ than the structures of the oxide glasses^25^, which limits the extraction of the detailed information from the diffraction data of metallic glasses. Besides, according to the relationship between the density and *S*(*Q*) diffraction peak position^26^, the density increase after the heat-treatment can be estimated to be ~0.68%. The increased density is not the aim of the schematic diagram^26^.


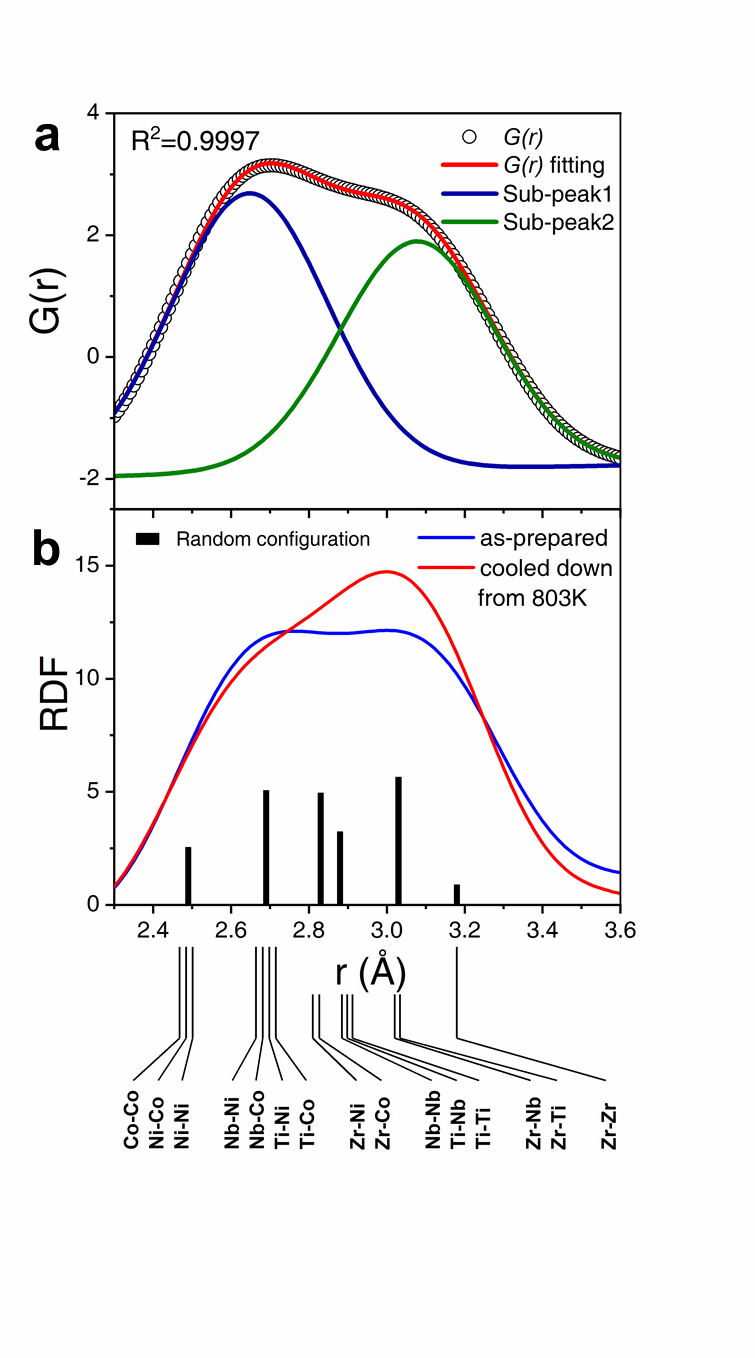


**Supplementary Figure 17. Fitting of the first peak of *G(r)* and the local structural changes in the first peak in radial distribution functions.** **a** High quality fitting of the first peak in *G(r)* of the as-prepared sample with two Gaussian sub-peaks. **b** Comparison of the first peak in radial distribution function of the as-prepared sample and the sample cooled down from 803 K to room temperature in the *in-situ* diffraction experiment. The 15 atomic pairs are grouped into six groups due to the similar bond lengths of some atomic pairs, and the heights of the bars correspond to the sum of of the atomic pairs in the same group.


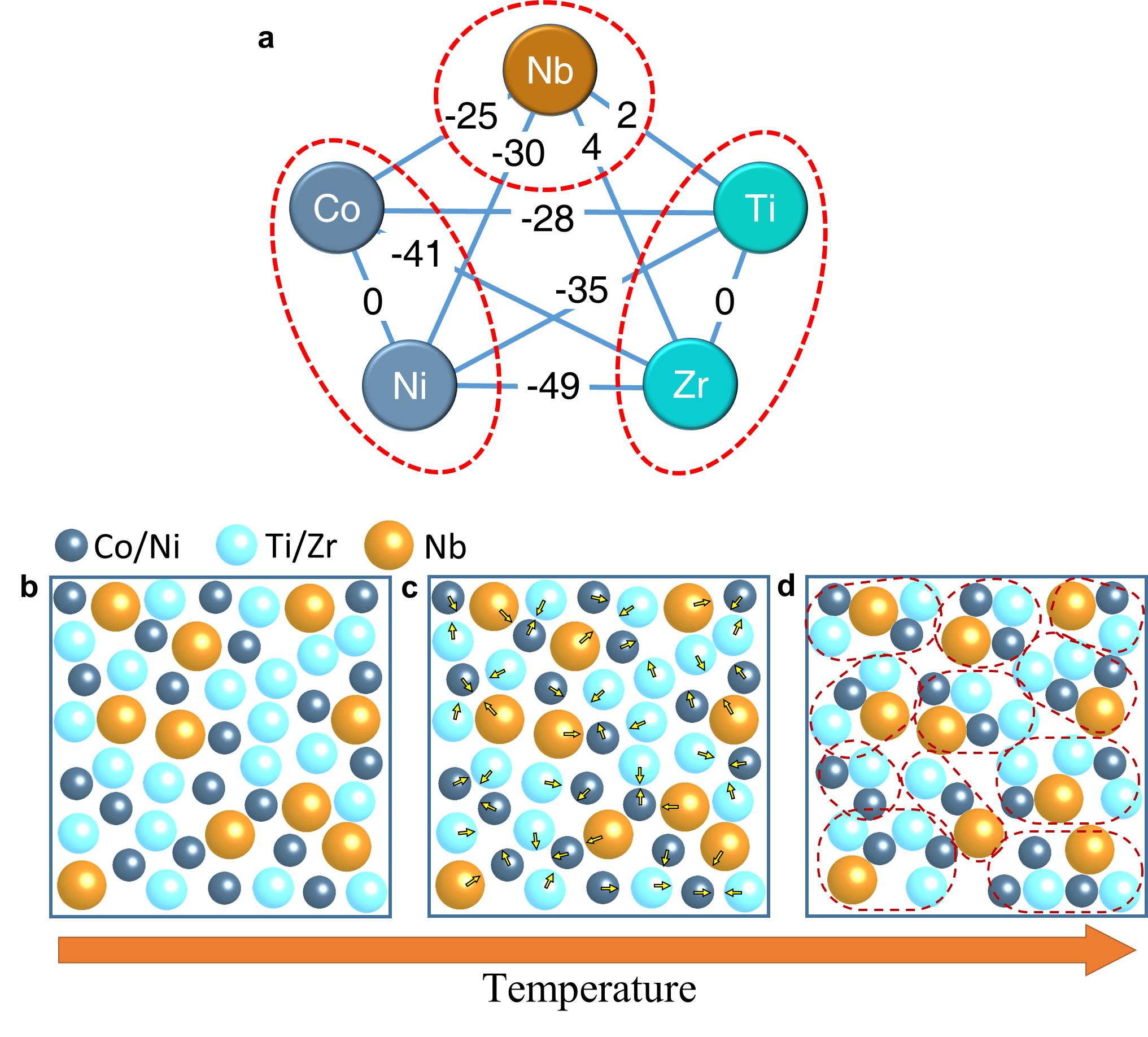


**Supplementary Figure 18.** **Schematic structural change of the phase transition. a** The mixing enthalpy of the atom pairs. The Co-Ni and Ti-Zr elements have similar chemical properties and zero mixing enthalpy, and thus they are denoted by the same type of atoms in **b**, **c** and **d** for simplicity and clarity. **b** The random distribution of the atoms in the as-prepared condition. **c** The moving trends of the atoms driven by the mixing entropy during the heating process. **d** The resulted more ordered structure after heating process. The closed dashed-lines are guide for the eye.

**Supplementary Note 8. Mechanical spectroscopy of the NbNiZrTiCo HEMG**

The storage modulus (Supplementary Fig. 1**9a**) shows a slight decrease during heating in the temperature range of the AEP and increases irreversibly during cooling. Empirically, the storage modulus is related to the short-range atomic bond. The increased modulus indicates a stronger atomic bond with higher bond energy^27^. The bond energy here is the energy required to break the atomic bond, which means that the transformation of an MG from a lower bond energy state to a higher bond energy state would release energy, corresponding to the AEP on the DSC curve. The irreversible increase in the storage modulus indicates higher bond energy, which is supposed to be caused by the change in short-range order. These results confirm the proposed irreversible glass-to-glass transition from a high-energy glass state to a low-energy glass state with a significant short-range structure change.


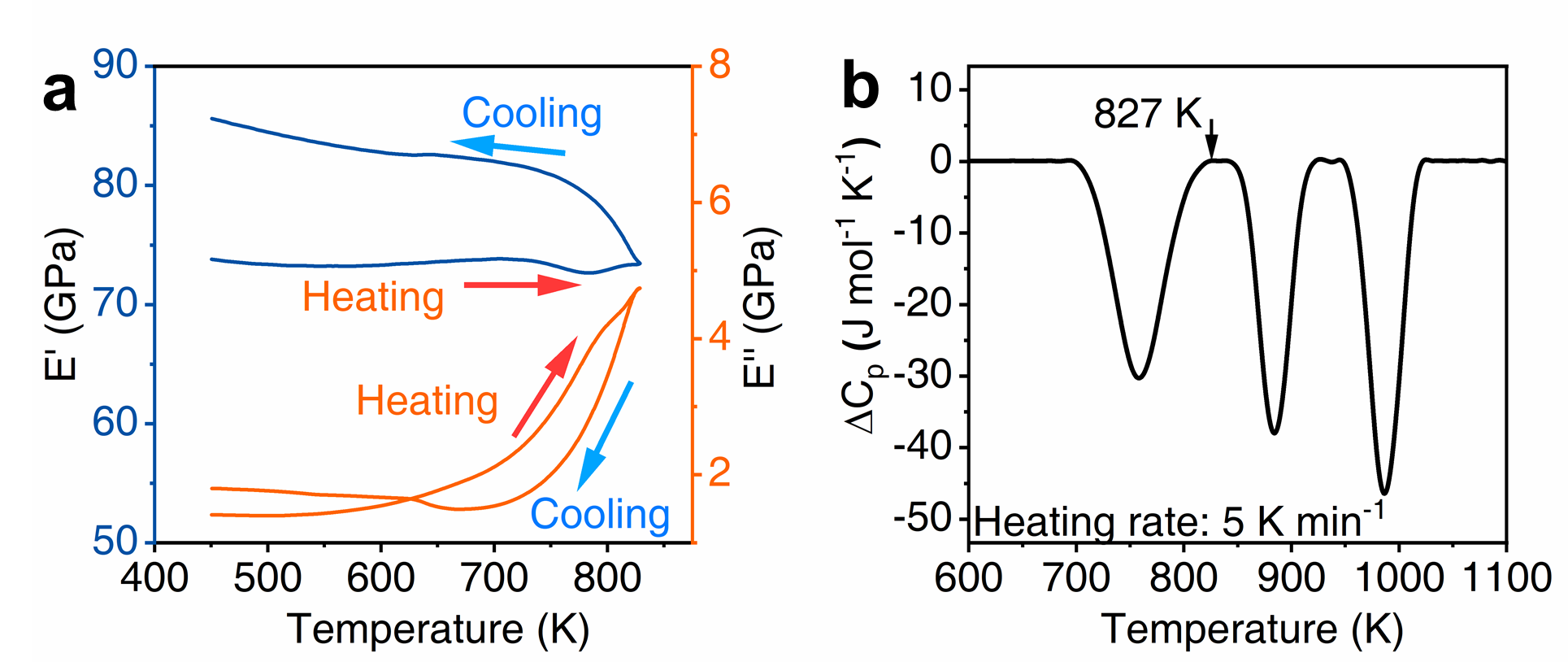


**Supplementary Figure 19. DMA results of the NbNiZrTiCo HEMG. a** Storage modulus *E’(T)* (dark blue line) and loss modulus *E’’(T)* (orange line) of the as-prepared NbNiZrTiCo HEMG during heating and cooling. **b** DSC curve of as-prepared NbNiZrTiCo HEMG with a heating rate of 5 K min^-1^.

**Experiment procedure:** Mechanical spectroscopy was performed on a TA Instruments Q800 dynamic mechanical analyzer. The test was performed in a tensile geometry applying a preload (static force) of 1.8 N. Oscillating strain of 1 μm amplitude was applied by loading and unloading dynamic force with 1 Hz frequency. The length of the ribbon was ~5 mm, which meant a relative strain oscillation amplitude of ~2*10^-4^. The sample was heated to 827 K and then cooled down to room temperature with heating and cooling rates of 5 K min^-1^.

**Supplementary Note 9.** **Nanoindentation analyses of the NbNiZrTiCo HEMG**

The typical load-displacement curves for the as-prepared and heat-treated samples are shown in Supplementary Fig. **20**, and the hardness and modulus extracted from the experiments are summarized in Supplementary Table **3**. The heat-treated sample exhibits 39.5 % higher hardness and 9.6 % higher modulus. The increased hardness and modulus indicate higher atomic bond energy, in agreement with the DMA results. It should be noted that the obtained modulus is different from the DMA results due to the different measuring methods.


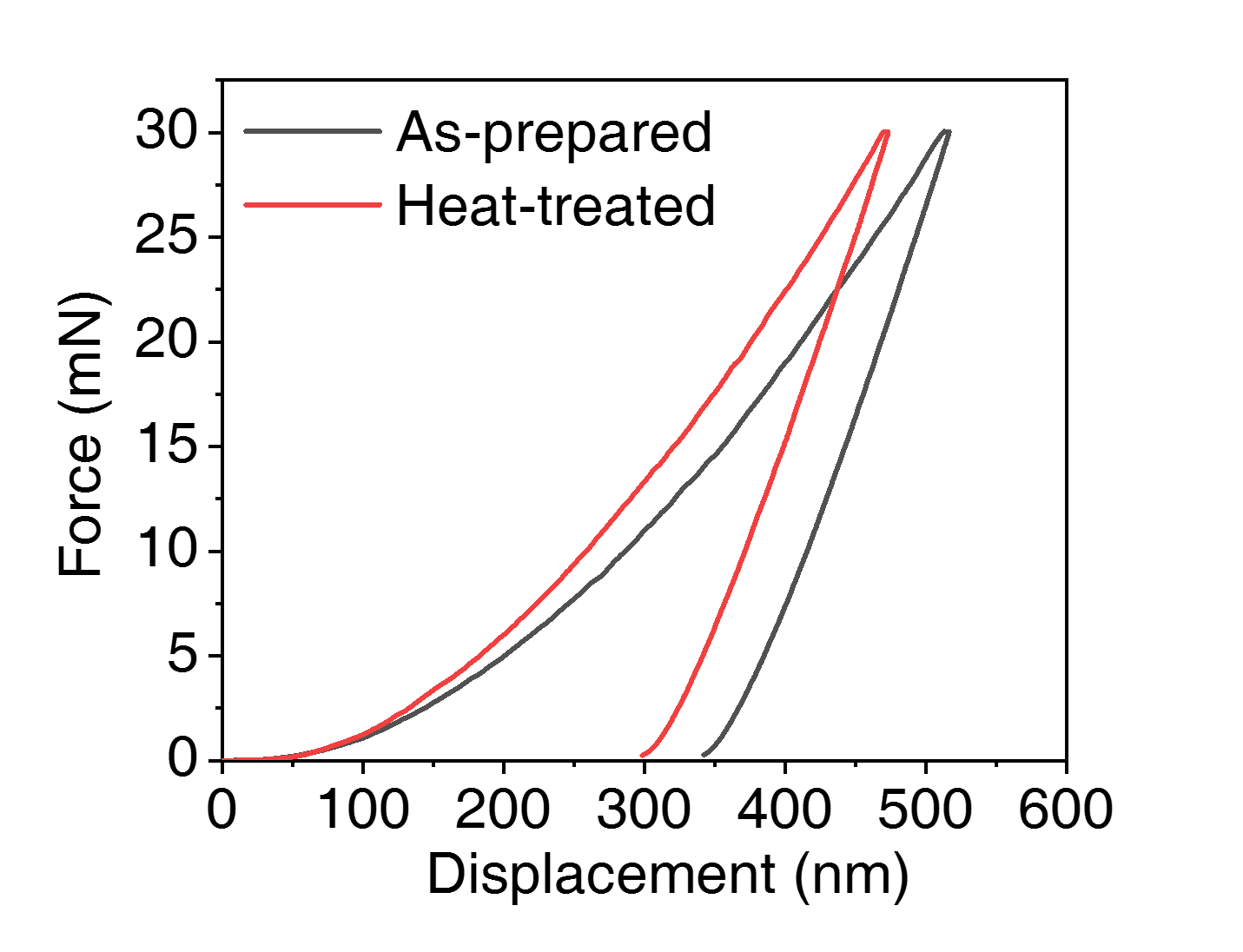


**Supplementary Figure 20. Typical load-displacement curves for the as-prepared and heat-treated samples.**

**Supplementary Table 3. Hardness and Young’s modulus of the as-prepared and heat-treated samples.**

| **Sample** | **Hardness (GPa)** | **Young’s Modulus (GPa)** |
| --- | --- | --- |
| As-prepared | 6.78 ± 0.11 | 86.3 ± 0.8 |
| Heat-treated | 9.46 ± 0.42 | 94.6 ± 0.9 |

**Experiment procedure:** Nanoindentation analyses on the as-prepared and heat-treated samples were performed using a Ti-900 nanoindenter (Hysitron Inc., USA) with a diamond Berkovich indenter (tip radii: 255 nm) at room temperature. 6 indents were made for each sample with the adjacent indents separated by at least 10 μm to avoid the overlap of the plastic deformation area. During each indentation test, the load was imposed with a constant loading rate of 1000 μN/s until the maximum load of 30 mN was reached. The maximum load was held for 5 s before unloading with 1000 μN s^-1^.

**Supplementary Note 10. Nanoindentation analyses of** **two additional HEMGs with glass-to-glass transitions**

Nanoindentation analyses were performed on the as-prepared and heat-treated NbNiZrTiCu and NbNiZrTiCoCu HEMGs. The hardness and modulus results are summarized in supplementary Table **4**. All three heat-treated samples exhibit higher hardness and modulus than the corresponding as-prepared samples, which is similar to the results of the NbNiZrTiCo HEMG.

**Supplementary Table 4. Hardness and Young’s modulus of the as-prepared and heat-treated NbNiZrTiCu and NbNiZrTiCoCu HEMGs.**

| **Sample** | **Hardness (GPa)** | **Young’s Modulus (GPa)** |
| --- | --- | --- |
| As-prepared NbNiZrTiCu | 9.21 ± 0.22 | 86.3 ± 0.8 |
| Heat-treated NbNiZrTiCu | 11.69 ± 0.51 | 114.9 ± 2.5 |
| As-prepared NbNiZrTiCoCu | 9.45 ± 0.18 | 109.3 ± 2.3 |
| Heat-treated NbNiZrTiCoCu | 11.36 ± 0.41 | 123.0 ± 3.2 |

**Experiment procedure:** Nanoindentation analyses on the as-prepared and heat-treated samples were performed using a Ti-900 nanoindenter (Hysitron Inc., USA) with a diamond Berkovich indenter (tip radii: 255 nm) at room temperature. 6 indents were made for each sample with the adjacent indents separated by at least 10 μm to avoid the overlap of the plastic deformation area. During each indentation test, the load was imposed with a constant loading rate of 1000 μN s^-1^ until the maximum load of 30 mN was reached. The maximum load was held for 5 s before unloading with 1000 μN s^-1^.

**Supplementary Note 11. Thermal stability of the two additional HEMGs with glass-to-glass transitions**

Isothermal DSC analyses are performed to reveal the time dependence of the stability of the heat-treated NbNiZrTiCu and NbNiZrTiCoCu HEMGs. It is found that these HEMGs remain amorphous after annealed at *T*_g_ + 10 K (NbNiZrTiCu, Supplementary Figs. **21a, b** and **c**) or *T*_g_ - 10 K (NbNiZrTiCoCu, Supplementary Figs. **21d, e** and **f**) for 2 hours. These results show that the HEMGs possess excellent thermal stability, and indicate that the glass-to-glass transition may function as a universally effective pathway to obtain metallic glasses with excellent thermal stability.

**
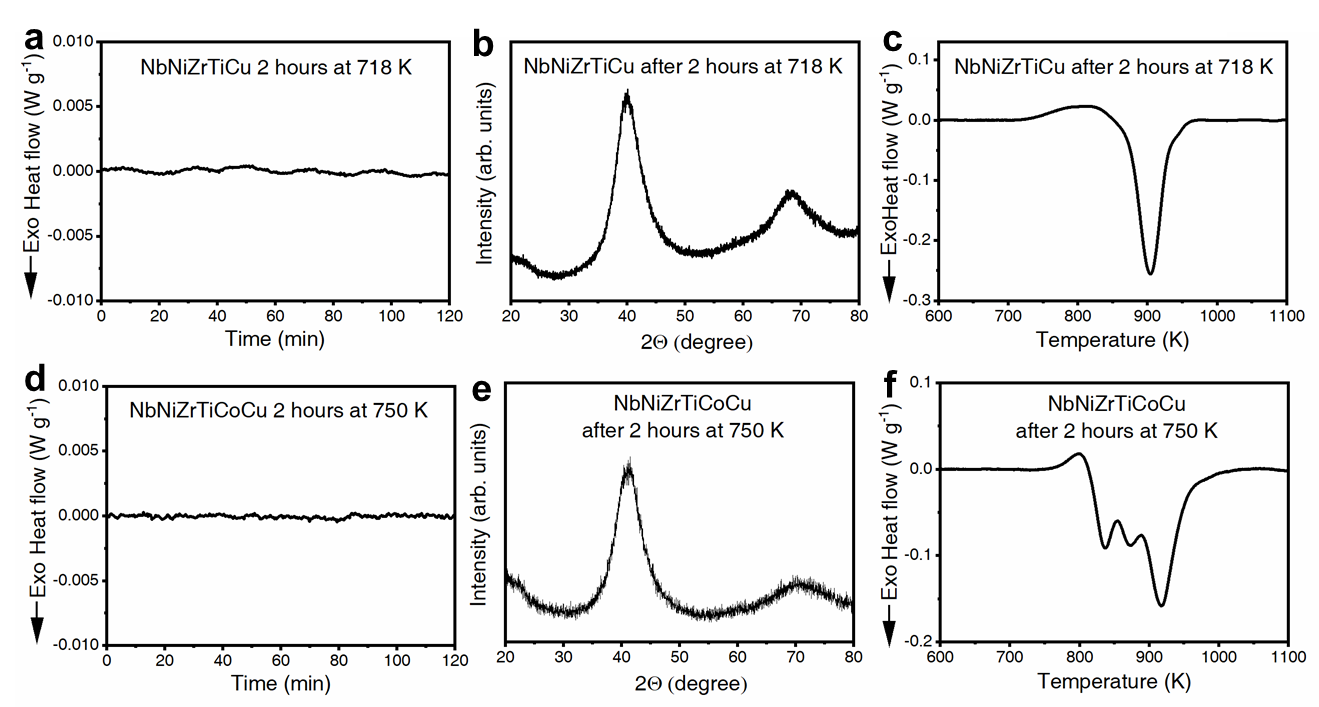
**

**Supplementary Figure 21. Isothermal DSC curves, X-ray diffraction analyses and DSC curves of the** **NbNiZrTiCu and NbNiZrTiCoCu alloys.** **a** Isothermal DSC curves of the heat-treated NbNiZrTiCu sample annealed at *T*_g_ + 10 K (718 K) for 2 hours. **b** XRD curve of the annealed NbNiZrTiCu sample. **c** DSC curve of the annealed NbNiZrTiCu sample. (d-f) Same as (a-c) but for the NbNiZrTiCoCu HEMG annealed at *T*_g_ - 10 K (750 K) for 2 hours.

**Experiment procedure:** DSC curves were measured using Al_2_O_3_ crucibles with a synchronous thermal analyzer (STA-449 F3, NETZSCH, Germany) under a high-purity argon atmosphere. The weight of each tested sample was ~20 mg. For isothermal scans experiments, the heat-treated samples were heated to *T_g_* + 10 K (NbNiZrTiCu) or *T_g_* - 10 K (NbNiZrTiCoCu) with a heating rate of 20 K min^-1^ and kept for 2 hours. Then, the samples were cooled down to room temperature and followed by a DSC scan with a heating rate of 20 K min^-1^. To highlight the phase transitions, the corresponding DSC curve of the crystallized HEMG was subtracted. The XRD analyses were performed using an X-ray diffractometer (D/max-RB, Rigaku Inc., Japan) with Cu *Kα* radiation (wavelength 1.5406 Å) at room temperature. The XRD experiments were performed using a *θ-2θ* mode with a scanning rate of 3 degrees min^-1^.

**Supplementary Note 12. Verification of the data process by the PDFgetX3 software**

The PDFgetX3 software was released in 2013^28^ by the same developer group of the PDFgetX2 software^28,29^, trying to avoid the human factors in data analysis especially to get highly consistent results in batch process and have been widely applied. The PDFgetX3 software was used in our work mainly for its “batch process” function, which could process the massive *in-situ* diffraction data efficiently and consistently. To show the reliability of the PDFgetX3 software for the purpose of our work, we have compared the diffraction results of the as-prepared sample processed by the PDFgetX3, PDFgetX2 and GSASII software as shown in Supplementary Fig. **22**. We find that the use of PDFgetX3 software does not have a visible influence on the peak shapes and peak positions of the results. We found that the peak intensity of data obtained from PDFgetX3 is always slightly lower than those from PDFgetX2 by a constant factor, which is consistent with the reports from literature^28,30^. Therefore, by multiplying a constant, the *G(r)* and *S(Q)* curves of the as-prepared sample processed by PDFgetX2 and PDFgetX3 coincide with each other very well (Supplementary Figs. **22c** and **d**). Therefore, the peak positions and shapes are independent of the use of PDFgetX2 or PDFgetX3 software. Besides, the low-r regions of the *G(r)* curves below the first peak show similar slopes and oscillation amplitudes, which are also signs of the reasonable quality of the experimental data^25,31^. We have also compared the diffraction results processed by the PDFgetX3 and GSASII software (Supplementary Figs. **22e**-**h**) and find similar results.

In addition, we also checked the diffraction data at the highest measurement temperature (803 K) using the PDFgetX2, PDFgetX3, and GSASII software, and we found the coefficient to match intensity is almost constant and independent of the temperature changes (Supplementary Fig. **23**), which means the relative change of peak intensity with varying temperatures is also reliable. These comparisons between different software evidence the validity of the data processed by PDFgetX3 in our work focusing on the peak shapes and positions rather than absolute overall intensity.

**
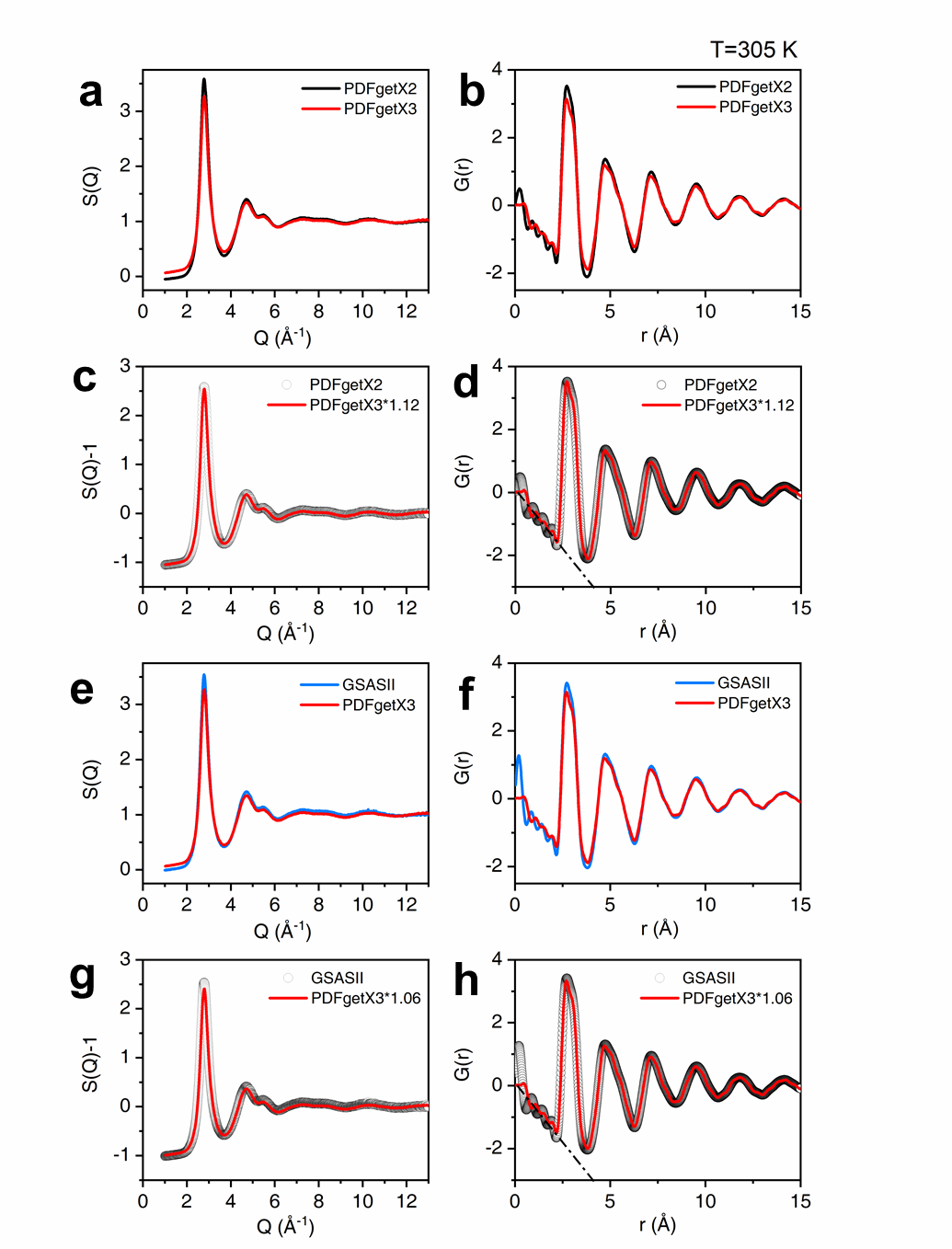
**

**Supplementary Figure 22. Comparison of diffraction data of as-prepared sample processed by the PDFgetX2, PDFgetX3, and GSASII software.** **a** *S(Q)* results processed by PDFgetX2 and PDFgetX3 software. **b** *G(r)* results processed by PDFgetX2 and PDFgetX3 software. **c** *S(Q)-1* results processed by PDFgetX2 and PDFgetX3 software with the PDFgetX3 result multiplied by a constant of 1.12. **d** *G(r)* results processed by PDFgetX2 and PDFgetX3 software with the PDFgetX3 result multiplied by a constant of 1.12. **e** *S(Q)* results processed by PDFgetX3 and GSASII software. **f** *G(r)* results processed by PDFgetX3 and GSASII software. **g** *S(Q)-1* results processed by GSASII and PDFgetX3 software with the PDFgetX3 result multiplied by a constant of 1.06. **h** *G(r)* results processed by GSASII and PDFgetX3 software with the PDFgetX3 result multiplied by a constant of 1.06. These results demonstrate that the *S(Q)* and *G(r)* data processed by the PDFgetX2, PDFgetX3 and GSASII software have slightly different intensities, while no significant difference in the peak position and peak shape is observed.


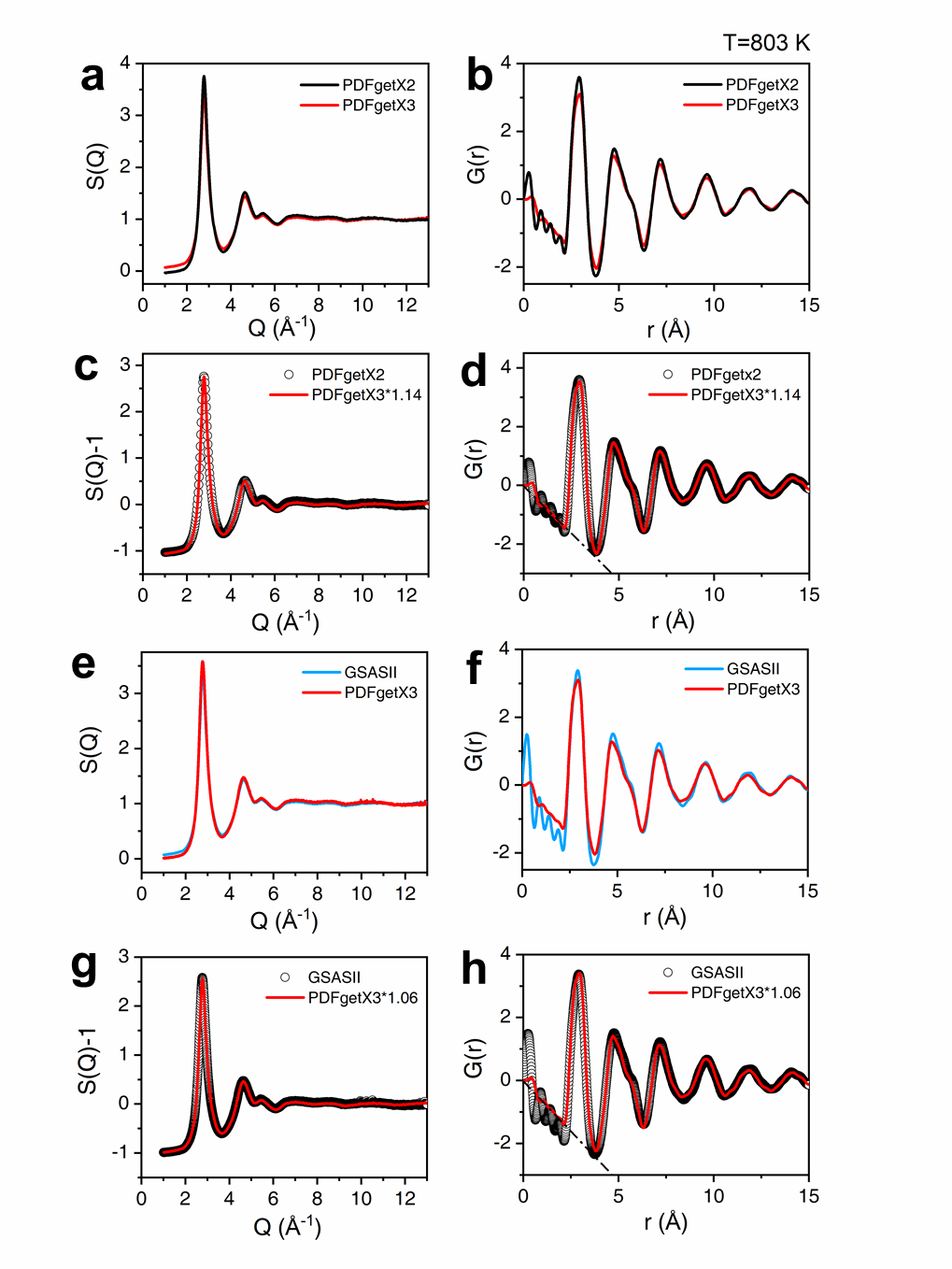


**Supplementary Figure 23. Comparison of diffraction data of sample at 803 K processed by the PDFgetX2, PDFgetX3, and GSASII software.** **a** *S(Q)* results processed by PDFgetX2 and PDFgetX3 software. **b** *G(r)* results processed by PDFgetX2 and PDFgetX3 software. **c** *S(Q)-1* results processed by PDFgetX2 and PDFgetX3 software with the PDFgetX3 result multiplied by a constant of 1.12. **d** *G(r)* results processed by PDFgetX2 and PDFgetX3 software with the PDFgetX3 result multiplied by a constant of 1.12. **e** *S(Q)* results processed by PDFgetX3 and GSASII software. **f** *G(r)* results processed by PDFgetX3 and GSASII software. **g** *S(Q)-1* results processed by GSASII and PDFgetX3 software with the PDFgetX3 result multiplied by a constant of 1.06. **h** *G(r)* results processed by GSASII and PDFgetX3 software with the PDFgetX3 result multiplied by a constant of 1.06. These results demonstrate that the *S(Q)* and *G(r)* data processed by the PDFgetX2, PDFgetX3 and GSASII software have slightly different intensities, while no significant difference in the peak position and peak shape is observed.


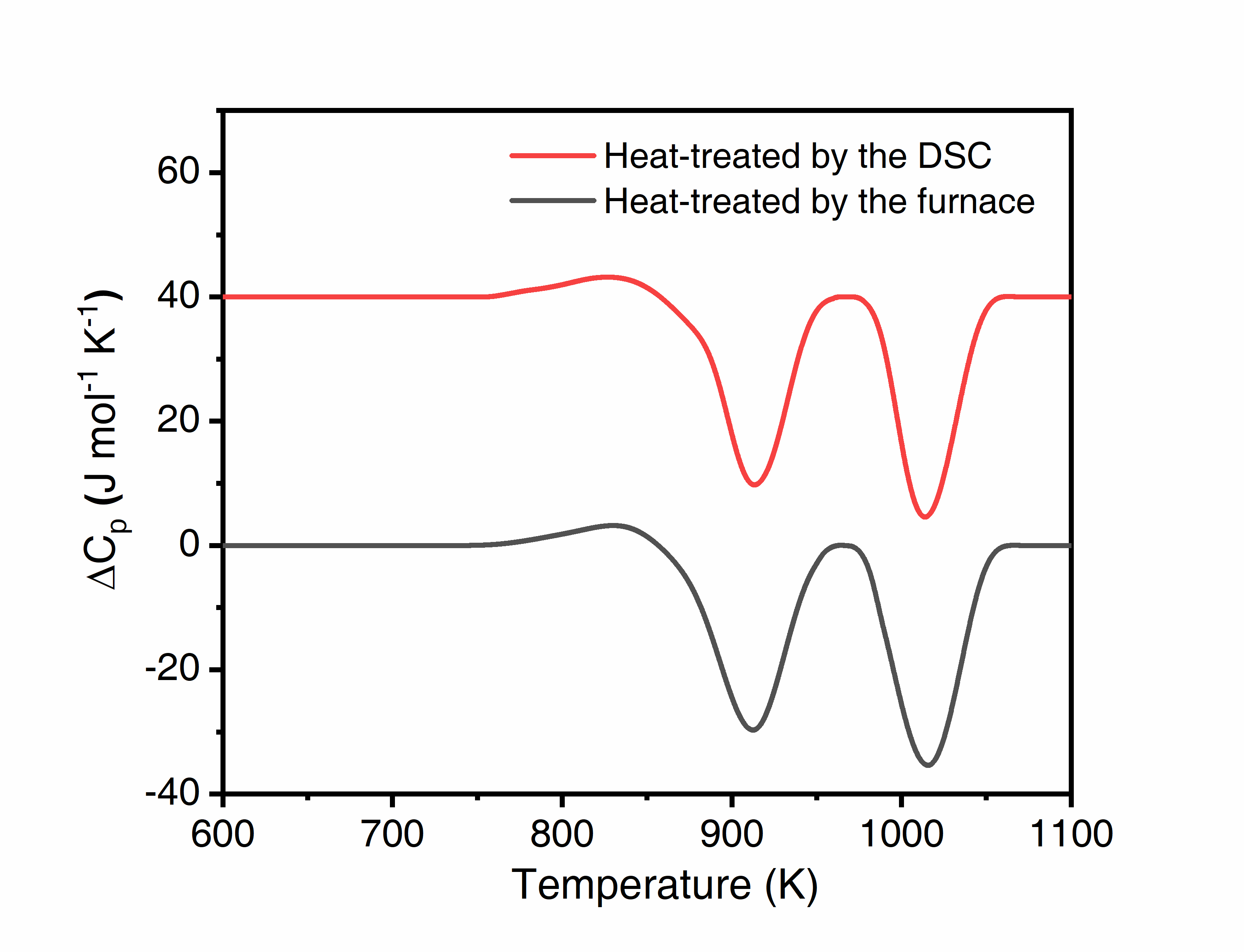


**Supplementary Figure 24. DSC curves of NbNiZrTiCo samples heat-treated by furnace and DSC.**

**Supplementary References**

1 Hu, H., Chen, L., Wang, X., Cao, Q. & Jiang, J. Formation of Ni–Nb–Zr–X (X= Ti, Ta, Fe, Cu, Co) bulk metallic glasses. *J. Alloys Compd.* **460**, 714-718 (2008).

2 Zhao, S. F., Yang, G. N., Ding, H. Y. & Yao, K. F. A quinary Ti–Zr–Hf–Be–Cu high entropy bulk metallic glass with a critical size of 12 mm. *Intermetallics* **61**, 47-50 (2015).

3 Ding, H. Y., Shao, Y., Gong, P., Li, J. F. & Yao, K. F. A senary TiZrHfCuNiBe high entropy bulk metallic glass with large glass-forming ability. *Mater. Lett.* **125**, 151-153 (2014).

4 Takeuchi, A. *et al.* Pd_20_Pt_20_Cu_20_Ni_20_P_20_ high-entropy alloy as a bulk metallic glass in the centimeter. *Intermetallics* **19**, 1546-1554 (2011).

5 Gao, X. Q. *et al.* High mixing entropy bulk metallic glasses. *J. Non-Cryst. Solids* **357**, 3557-3560 (2011).

6 Bizhanova, G., Li, F., Ma, Y., Gong, P. & Wang, X. Development and crystallization kinetics of novel near-equiatomic high-entropy bulk metallic glasses. *J. Alloys Compd.* **779**, 474-486 (2019).

7 Xu, Y., Li, Y., Zhu, Z. & Zhang, W. Formation and properties of Fe_25_Co_25_Ni_25_(P, C, B, Si)_25_ high-entropy bulk metallic glasses. *J. Non-Cryst. Solids* **487**, 60-64 (2018).

8 Zhao, S. F. *et al.* Pseudo-quinary Ti_20_Zr_20_Hf_20_Be_20_(Cu_20-x_Ni_x_) high entropy bulk metallic glasses with large glass forming ability. *Mater. Des.* **87**, 625-631 (2015).

9 Qi, T. *et al.* Soft magnetic Fe_25_Co_25_Ni_25_(B, Si)_25_ high entropy bulk metallic glasses. *Intermetallics* **66**, 8-12 (2015).

10 Gong, P., Li, F., Deng, L., Wang, X. & Jin, J. Research on nano-scratching behavior of TiZrHfBeCu(Ni) high entropy bulk metallic glasses. *J. Alloys Compd.* **817** (2020).

11 Wu, L., Zhao, Y., Li, J.-j., Wu, J.-l. & Zhang, B. Correlation between mechanical and thermodynamic properties for La-Ce-Ni-Cu-Al high-entropy metallic glasses. *J. Iron Steel Res. Int.* **25**, 658-665 (2018).

12 Sheng, W. *et al.* Amorphous microwires of high entropy alloys with large magnetocaloric effect. *Intermetallics* **96**, 79-83 (2018).

13 Huo, J. *et al.* High-entropy bulk metallic glasses as promising magnetic refrigerants. *J. Appl. Phys.* **117** (2015).

14 Li, J. *et al.* Distinct spin glass behavior and excellent magnetocaloric effect in Er_20_Dy_20_Co_20_Al_20_RE_20_ (RE = Gd, Tb and Tm) high-entropy bulk metallic glasses. *Intermetallics* **96**, 90-93 (2018).

15 Pang, C. M. *et al.* Effect of Dy, Ho, and Er substitution on the magnetocaloric properties of Gd-Co-Al-Y high entropy bulk metallic glasses. *J. Alloys Compd.* **827** (2020).

16 Pang, C. M. *et al.* Effect of Yttrium addition on magnetocaloric properties of Gd-Co-Al-Ho high entropy metallic glasses. *J. Non-Cryst. Solids* **549** (2020).

17 Xue, L., Shao, L., Luo, Q. & Shen, B. Gd_25_RE_25_Co_25_Al_25_ (RE = Tb, Dy and Ho) high-entropy glassy alloys with distinct spin-glass behavior and good magnetocaloric effect. *J. Alloys Compd.* **790**, 633-639 (2019).

18 Wu, K. *et al.* Magnetocaloric effect of Fe_25_Co_25_Ni_25_Mo_5_P_10_B_10_ high-entropy bulk metallic glass. *J. Magn. Magn. Mater.* **489** (2019).

19 Lefebvre, W., Vurpillot, F. & Sauvage, X. *Atom probe tomography: put theory into practice*. (Academic Press, London, 2016).

20 Moody, M. P., Stephenson, L. T., Ceguerra, A. V. & Ringer, S. P. Quantitative binomial distribution analyses of nanoscale like-solute atom clustering and segregation in atom probe tomography data. *Microsc. Res. Tech.* **71**, 542-550 (2008).

21 Bednarcik, J. *et al.* Thermal expansion of a La-based bulk metallic glass: insight from *in situ* high-energy x-ray diffraction. *J. Phys. Condens. Matter* **23**, 254204 (2011).

22 Qu, D. D. *et al.* On the Atomic Anisotropy of Thermal Expansion in Bulk Metallic Glass. *Adv. Eng. Mater.* **13**, 861-864 (2011).

23 Georgarakis, K. *et al.* Probing the structure of a liquid metal during vitrification. *Acta Mater.* **87**, 174-186 (2015).

24 Yue, X. X., Inoue, A., Liu, C. T. & Fan, C. The Development of Structure Model in Metallic Glasses. *Mat. Res.* **20**, 326-338 (2017).

25 Wright, A. C. Neutron scattering from vitreous silica. V. The structure of vitreous silica: What have we learned from 60 years of diffraction studies? *J. Non-Cryst. Solids* **179**, 84-115 (1994).

26 Yavari, A. R. *et al.* Excess free volume in metallic glasses measured by X-ray diffraction. *Acta Mater.* **53**, 1611-1619 (2005).

27 Buschow, K. H. J. *et al.* *Encyclopedia of Materials: Science and Technology*. 2nd edn, (Elsevier, Amsterdam, 2001).

28 Juhas, P., Davis, T., Farrow, C. L. & Billinge, S. J. L. PDFgetX3: a rapid and highly automatable program for processing powder diffraction data into total scattering pair distribution functions. *J. Appl. Crystallogr.* **46**, 560-566 (2013).

29 Qiu, X., Thompson, J. W. & Billinge, S. J. L. PDFgetX2: a GUI-driven program to obtain the pair distribution function from X-ray powder diffraction data. *J. Appl. Crystallogr.* **37**, 678 (2004).

30 Peterson, P. F., Bozin, E. S., Proffen, T. & Billinge, S. J. L. Improved measures of quality for the atomic pair distribution function. *J. Appl. Crystallogr.* **36**, 53-64 (2003).

31 Wright, A. C. Neutron and X-Ray Amorphography. *J. Non-Cryst. Solids* **106**, 1-16 (1988).
